# Excess molybdenum impairs growth, nitrogen metabolism and nutrient translocation of soybean in *Bradyrhizobium* symbiosis, with parallels to tungsten stress

**DOI:** 10.3389/fpls.2026.1905971

**Published:** 2026-08-07

**Authors:** Julian Preiner, Irene Steccari, Eva Oburger, Stefanie Wienkoop

**Affiliations:** 1 Division of Molecular Systems Biology, Department of Functional and Evolutionary Ecology, University of Vienna, Vienna, Austria; 2 Institute of Soil Research, Department of Forest and Soil Sciences, University of Natural Resources and Life Sciences Vienna, Tulln, Austria

**Keywords:** abiotic stress, metabolomics, molybdenum toxicity, nitrogen metabolism, proteomics, rhizobia symbiosis, soybean (*Glycine max*), tungsten toxicity

## Abstract

Molybdenum (Mo) serves an important biological role as part of cofactors of various enzymes in all domains of life. In plants, it is critical for nitrogen metabolism and integral to rhizobial enzymes such as nitrogenase, which is essential for plant-rhizobia symbiosis. However, like other transition metals, Mo can be toxic at excess concentrations. Its chemical analog tungsten (W) has no biological function in eukaryotes and is known to be toxic, primarily by inhibiting molybdoenzymes. Previously, we found that soybean (*Glycine max*) in symbiosis with N_2_-fixing rhizobia (*Bradyrhizobium japonicum*) (N fix plants) had greater capacity to synthesize protective compounds in response to W-induced stress than KNO_3_-fertilized (N fed) plants. This shotgun metabolomic and proteomic study investigates the response of N fed and N fix plants to excess Mo (0.5 mM Na_2_MoO_4_), whether the metabolic plasticity observed upon W-stress is also present in Mo-exposed N fix plants and the key drivers of this plasticity. Our results show that, similar to W, excess Mo elicits a strong metabolic response in symbiotic soybean plants, disrupting plant growth, photosynthesis, nitrogen metabolism and the translocation of essential nutrients including Fe, Cu, Mn and S. Furthermore, excess Mo elicits a profile of protective compounds that is qualitatively similar to, but quantitatively distinct from, that seen under W stress. Notably, while this defense response was more pronounced in symbiotic plants (N fix) under W and Mo stress, it did not result in restored plant growth. Together, these findings demonstrate that the deployment of protective compounds is largely independent of whether the excess metal is essential for plant growth. The fact that this response is associated with the plant-rhizobia symbiosis highlights the importance of biotic interactions for shaping a plant’s chemical defense against abiotic stress.

## 1 Introduction

Molybdenum (Mo) is an essential trace element that serves an important biological role as a component of cofactors of various enzymes in all domains of life. In plants, molybdenum cofactors (Moco) are present in five key enzymes catalyzing assimilatory and catabolic processes such as nitrate reductase (NR), xanthine dehydrogenase/oxidase, aldehyde oxidase, sulfite oxidase and the mitochondrial amidoxime–reducing component (Kaiser et al., 2005; Tejada-Jiménez et al., 2013). The bacterial enzyme nitrogenase is an exception in this respect, as it contains a distinct FeMo-cofactor that catalyzes the redox reaction to convert and thus fix elemental N_2_ into ammonium (Bittner, 2014). Due to its central role in these enzymes, Mo availability is indispensable for nitrogen assimilation, purine catabolism, sulfite detoxification and abscisic acid biosynthesis in plants and thus for optimal plant growth, development and productivity. However, nutritional demand differs considerably between plant species, with nutrient-rich crop plants such as leguminous plants and Brassicaceae having a higher Mo demand compared to cereal crops and forage crops (Anjum et al., 2015; Mengel et al., 2001).

Leguminous plants are of particular interest in studying Mo metabolism, as they rely not only on the plant’s own molybdoenzymes, but also on the bacterial nitrogenase for optimal nitrogen nutrition. Soybeans rely on an additional Mo-containing enzyme, xanthine dehydrogenase, which mediates the production of allantoin and allantoic acid, the major transport form of N in subtropical legumes (Mengel et al., 2001). Agronomically important legumes such as soybeans (*Glycine max*) cover approximately 50-60% of their N demand by biological nitrogen fixation (BNF) (Salvagiotti et al., 2008), which represents a sustainable and cost-effective alternative to synthetic nitrogen fertilizers (Tejada-Jiménez et al., 2018). As legume-rhizobia symbioses introduce approximately 33–46 million tons of N annually into agricultural soils (Herridge et al., 2008), adequate supply and bioavailability of Mo in soils are of agricultural and ecological relevance (Bursakov et al., 2023).

Plants are comparatively tolerant to elevated Mo concentrations; nevertheless, similarly to other nutrients, it has been found that there is a narrow margin between Mo deficiency and toxicity (Tejada-Jiménez et al., 2013). Although symbiotically grown leguminous plants have a higher Mo demand (Mengel et al., 2001), negative effects of excess molybdenum on the legume-rhizobia symbiosis are documented. In hairy vetch, Mo concentrations higher than 1 mg kg^-1^ led to a deterioration of nodule structure and reduced nitrogenase activity (Alam et al., 2015). Yang et al. (2020) reported a significant inhibition of N_2_ fixation, nodule mass and nodule number, as well as plant biomass production. These effects were accompanied by structural changes and increased antioxidative enzyme activity in symbiotically grown soybeans treated with 100 mg kg^-1^ Mo nanomaterials (Mo, MoO_2_, MoO_3_). Although uptake, translocation and behavior of these nanomaterials within the plant were not investigated in this study, and these nanomaterials cannot be directly compared to sodium molybdate, the results are nevertheless noteworthy with respect to the reported Mo tissue concentrations and the physiological effects described. In young pea plants, Kevresan et al. (2001) found that molybdenum concentrations of 0.01 mM and 1 mM led to a significant growth retardation and a reduction of dry matter by 35% and 50%, respectively. In sum, these findings indicate that excess Mo can disrupt plant growth, nitrogen metabolism and symbiotic function in legumes. However, the underlying molecular and metabolic mechanisms and how plants respond to excess Mo, remain poorly understood.

Molybdenum is primarily taken up in the form of molybdate (MoO_4_^2-^), with soil properties such as pH being key determinants of its bioavailability (Mengel et al., 2001; Tejada-Jiménez et al., 2018). In different plant species, specific MoO_4_^2-^ transporters such as MOT1 and MOT2 have been identified to mediate uptake and long-distance transport from roots to shoots (Tejada-Jiménez et al., 2018). Excess Mo appears to be primarily stored in vacuoles of peripheral parenchyma cells (Hale et al., 2001). Beyond these specific Mo transporters, as well as Moco- and nitrogenase FeMo-cofactor biosynthesis, the intracellular fate of Mo - including chelation, sequestration, organ-level partitioning and export - remains only partially understood (Schwarz et al., 2009; Tejada-Jiménez et al., 2013). This is, however, particularly relevant in the context of potential Mo toxicity, as the buffer capacity within the cell may determine the difference between adequate supply and cytotoxic accumulation.

Molybdenum shares certain chemical properties with the transition element tungsten (W) (Bevers et al., 2009). However, while molybdenum serves an important biological role as part of cofactors of various enzymes, tungsten is only utilized in certain anaerobic bacteria and archaea and has no known biological function in eukaryotes (Bevers et al., 2009; Hille, 2002; Kletzin and Adams, 1996). Due to their chemical similarities, tungsten is readily taken up by plants and competes with Mo for incorporation into the molybdopterin cofactors of molybdoenzymes (Deng et al., 1989; Xiong et al., 2012). These W-substituted cofactors have been shown to exhibit reduced catalytic activity, which results in the disruption of central metabolic pathways, growth retardation and reduced photosynthetic activity (Adamakis et al., 2012; Bevers et al., 2009). Previous studies demonstrated that W-toxicity is not limited to the inhibition of molybdoenzymes, but that it leads to a broader metabolic reprogramming as well as a disruption of cellular redox homeostasis (Adamakis et al., 2014; Adamakis and Eleftheriou, 2019; Preiner et al., 2019, 2024). Furthermore, we found that symbiotically grown soybeans exhibit greater systemic plasticity in their stress response to tungsten and are able to accumulate a range of protective compounds such as phenols, polyamines, gluconic acid and amino acids including proline (Preiner et al., 2024).

In light of the chemical similarity between Mo and W, the aim of this study is to determine whether excess concentrations of the essential nutrient molybdenum trigger a comparable metabolic defense response to that previously reported for tungsten. Furthermore, we aim to investigate whether the metabolic plasticity observed in symbiotic plants under W-stress is equally induced under excess Mo conditions and to identify key drivers of this plasticity. Finally, we examine whether this increased investment in protective compounds and metabolic readjustment translates to enhanced stress tolerance against the two transition metals. The study thereby provides new insights into how these chemically analogous transition metals affect plant metabolic processes and the role that bacterial symbionts play in their host’s abiotic stress response.

## 2 Materials and methods

### 2.1 Plant culture and harvest

A detailed account of cultivation as well as a description of growth conditions of soybean plants (*Glycine max* cv. Primus obtained from Die SAAT Austria) can be found in Preiner et al. (2024). In brief, surface-sterilized seeds were sown into acid-washed pots containing a substrate mixture of vermiculite and perlite in a 5/3 (v/v) ratio and watered with a modified half-strength Hoagland nutrient solution (N-free, pH 7.2, conductivity of 1066–1160 μS cm^−1^ between control and 0.5 mM Mo) (Hoagland and Arnon, 1950). Half of the sterilized seeds were inoculated by soaking them in a 1:10 diluted commercial *Bradyrhizobium japonicum* inoculant (Radicin Soja, Die SAAT Austria) and were subsequently supplied with 0.25 mM KNO_3_ for two weeks (hereafter referred to as N fix). Additionally, 1.5 mL of the diluted inoculant was directly applied to the pots after planting the seeds. The other half was supplied with 10 mM KNO_3_ throughout the experiment and served as a non-symbiotic control (referred to as N fed). Plants were grown in eight biological replicates. Three weeks after germination (21 d), half the plants of each N-regime were exposed to 0.5 mM sodium molybdate (Na_2_MoO_4_). This was done to ensure that symbiosis was established and nodules were fully developed before induction of the excess Mo treatment. After two weeks of Mo-exposure, four biological replicates per treatment were harvested for metabolomic and proteomic analysis with LN_2_ and stored at -80 °C. The remaining four replicates were used for biomass assessment and determination of nutrient contents via Inductively Coupled Plasma Optical Emission Spectroscopy (ICP-OES) analysis.

### 2.2 Chlorophyll fluorescence measurements

Pulse-amplitude-modulated (PAM) chlorophyll fluorescence measurements were performed with a MINI-PAM-II/B fluorometer (Heinz Walz GmbH) as described in Preiner et al. (2024). In brief, the plants were dark-adapted for 15 min and then subjected to a rapid light curve (RLC) with increasing intensities of photosynthetically active radiation (PAR) in 20 s intervals. Calculations were performed with the WinControl-3 software (Heinz Walz GmbH).

### 2.3 Sequential extraction of metabolites and proteins

Metabolite and protein extraction was performed according to Preiner et al. (2024). In brief, 60 mg of finely ground frozen plant material were covered with 1 mL of ice-cold 80% acetone and subsequently vortexed and centrifuged at 10,000 × *g* for 10 minutes. The supernatant was collected and the extraction procedure repeated two times with 0.5 mL 80% acetone. The resulting pellet was then extracted in 1 mL 80% methanol, vortexed and incubated at 95 °C for 30 minutes. After centrifugation at 10,000 × *g* for 10 minutes, the pellet was then re-extracted in 80% ethanol, again heated at 95 °C for 30 minutes and centrifuged at 10,000 × *g* for 10 minutes to remove remaining metabolites. Subsequently, two aliquots per sample were prepared for metabolite analysis by pooling 1 mL of the acetone extract and 1 mL of the combined ethanol-methanol extracts. One aliquot was used for photometric determination of key metabolites; the second aliquot was spiked with internal standards (5 µl 1 mM phenyl-β-D-glucopyranoside and 10 mM pentaerythritol) and dried under N_2_ flow. Standards, quality control samples (QCs) and blanks were prepared accordingly. The pellets were frozen at -80°C for protein extraction.

#### 2.3.1 Photometric measurement of pigments, sugars, secondary metabolites and antioxidant capacity

The supernatant of the acetone extraction step was used for photometric estimation of photosynthetic pigments according to Lichtenthaler and Wellburn (1983). For all following photometric assays, a compound sample of supernatants from the acetone, ethanol and methanol extraction steps was used, as previously described. Determination of soluble carbohydrate content was adapted from Hansen and Møller (1975). Assays for total phenols and flavonoids, condensed tannins and antioxidant capacity (IC50) were performed according to Rebaya et al. (2015); Broadhurst and Jones (1978) and Chang et al. (2002). A detailed account of the assays as well as all changes made to the original protocols can be found in Preiner et al. (2024).

#### 2.3.2 GC-MS analysis

To comprehensively and efficiently profile and quantify a broad range of primary metabolites in a single analytical run, gas chromatography-mass spectrometry (GC-MS) was chosen over liquid chromatography-mass spectrometry (LC-MS). GC-MS is a robust technology that enables both targeted and untargeted profiling of a broad range of metabolites and benefits from the availability of comprehensive, standardized mass spectral libraries for reliable metabolite identification (Fiehn, 2016). Samples were prepared as described in Preiner et al. (2024). In summary, dry samples were derivatized by adding 20 μL of a 40 g l^−1^ methoxyamine hydrochloride in pyridine and incubating them at 30 °C for 90 min. Subsequently, 80 μL N-methyl-N-trimethylsilyl-trifluoroacetamide (MSTFA) was added followed by another 30 minutes at 37 °C for silylation. Samples were then centrifuged at 14,000 × *g* for 2 min, transferred to MS vials and measured in split 100 and split 5 mode with an Agilent 7890B GC coupled to a LECO Pegasus^®^ BT GC-TOFMS (LECO Corporation, Michigan, USA). Standards, quality control samples (QCs) and blank samples were prepared accordingly alongside the samples. As retention index (RI) markers, alkane analytical standards (C10-C40) (Sigma-Aldrich) were injected at the beginning and end of each batch. A detailed account of the parameters used for GC-MS analysis can be found in Preiner et al. (2024). In brief, the front inlet temperature was set to 230 °C for the entire run. The initial oven temperature was set to 70 °C for 1 min and was increased with a rate of 9 °C min^−1^ to a final temperature of 330 °C. This temperature was held for 8 min. The acquisition delay was set to 5 min and the acquisition rate was set to 10 spectra per second. The mass range was set from 50 to 600 *m/z*, the extraction frequency was set to 30 kHz and the detector voltage was set to 1,550 V. Raw data were processed with the LECO ChromaTOF^®^ software (LECO^®^ Corporation, MI, USA).

#### 2.3.3 Protein extraction and digestion

Protein extraction was performed according to Preiner et al. (2024). In brief, pellets obtained from the metabolite extraction were covered with 1 mL of protein extraction buffer [urea 8 M; 4-(2-hydroxyethyl)-1-piperazineethanesulfonic acid (HEPES) 50 mM, pH 7.8], vortexed and incubated at 4 °C for 60 min. After overnight precipitation at -20 °C in acetone with 0.5% β-mercaptoethanol, the pellets were washed three times with 1 mL ice-cold acetone and then air-dried at room temperature. The pellet was re-dissolved in buffer containing 8 M urea and 50 mM HEPES at pH 7.8 and protein content determined using a Bradford protein assay from Bio-Rad Laboratories.

Subsequently, protein content of samples was normalized to 10 μg protein, and samples were reduced and alkylated with 5 mM dithiothreitol (DTT) and 10 mM iodoacetamide (IAA). After diluting urea sample concentration by adding an equal volume of 100 mM ammonium bicarbonate (AmBic) in 20% acetonitrile (ACN) to 4 M, 0.1 μg Lys-C was added, and the samples were incubated at 30 °C for 5 h at 500 rpm in the dark (Niessen et al., 2006). Then urea concentration was again diluted from 4 M to 2 M by adding another volume of 10% acetonitrile containing 25 mM Ammonium Bicarbonate, 10 mM CaCl_2_, and 5 mM DTT. Thereafter, 3 μL of trypsin beads (Poroszyme, Applied Biosystems) were added, and samples were incubated for 14 h at 37 °C at 10 rpm in a rotating hybridization oven (Boekel Little Shot III). Finally, the digested proteins were desalted with Agilent Bond Elut OMIX C18 pipette-based solid-phase extraction stage tips (Agilent Technologies) and dried in a vacuum concentrator (ScanSpeed MaxiVac), and stored at -80 °C. A detailed description of the digestion procedure described above can be found in Preiner et al. (2024).

##### 2.3.3.1 LC-MS/MS measurement and protein identification

Dried digested protein samples were dissolved in 100 μL 2% acetonitrile (ACN) and 0.1% formic acid (FA), ultrasonicated for 15 seconds. Samples were then centrifuged at 4 °C for 10 minutes at 21,000 × *g* and transferred to mass spectrometry (MS) vials. Separation of peptides was performed with an ultra-high-performance liquid chromatography (uHPLC) system (Dionex Ultimate 3000) with a flow rate of 300 μl min^-1^. MS analysis was carried out with a Thermo Scientific Velos Pro ion trap and Thermo Scientific LTQ Orbitrap. Mass spectral data were analyzed using MaxQuant (2.0.3.1) as described in Preiner et al. (2019) using a combined FASTA file for *Glycine max* and *Bradyrhizobium japonicum* with 115,028 entries (http://www.uniprot.org/ accessed 25 April 2019). Instrument settings, measurement details and identification can be found in Preiner et al. (2024).

#### 2.3.4 Estimation of starch concentration

The pellet from the protein extraction was used to perform an enzymatic assay for photometric determination of starch content according to Nägele and Heyer (2013) as described in Preiner et al. (2019).

### 2.4 Acid digestion and elemental analysis

After the harvest, a subset of plants was processed and digested according to Preiner et al. (2024) for elemental analysis. Nutrient contents were analyzed with Inductively Coupled Plasma Optical Emission Spectroscopy (ICP-OES Optima 3000 XL Perkin Elmer). Translocation factors (TF) for individual nutrients and molybdenum were calculated by dividing tissue concentrations of shoots by concentrations in roots.

### 2.5 Statistical analysis and data mining

Data were only used for statistical analysis if values for at least three replicates in at least one treatment were recorded. When less than half of the values of one treatment were missing, values were imputed using the k-nearest neighbor algorithm. If more than half of the values of one treatment were missing, the smallest value detected throughout all samples divided by two (minimal value) was used to impute missing data points. Two samples of one treatment (Mo N fix root/leaf) were lost during sample processing (metabolite extraction) and thus were not imputed as they were considered systematically missing (n = 3-4).

Statistical analysis of data from photometric assays, GC-MS and ICP-OES as well as physiological data was performed with InfoStat software (InfoStat, RRID: SCR_014310; Di Rienzo et al., 2012). Data were analyzed using a linear mixed model and DGC *post hoc* test. If necessary, heteroscedasticity was corrected by selecting the varIdent model in InfoStat software. *P*-values smaller than 0.05 were considered significant.

An analysis of variance (ANOVA) followed by a Tukey HSD *post hoc* test was performed on protein data using R (R Core Team, 2021). Proteins were considered significantly changed when the change (ratio) between compared treatments was at least 2-fold, with a p-value below 0.05. P-value correction was performed using Benjamini-Hochberg to account for false positives due to multiple testing; adjusted *p*-values are provided in the summary tables in the supplement (**Supplementary Tables 5**, **S6**).

A principal component analysis (PCA) was performed with MetaboAnalyst 6.0 (Pang et al., 2024) using a log_2_ transformed data matrix created for roots as well as leaves. Each matrix contained all metabolites and significantly changed proteins that were detected in at least three out of four replicates of at least one treatment. Heatmaps were also created using MetaboAnalyst 6.0, Euclidean distance with complete linkage was used for clustering. PCA loadings for proteins can be found in **Supplementary Tables 4**, **S5**. Loadings for metabolites are provided in **Supplementary Tables 2**, **S3**.

GO enrichment analysis as well as annotation for molecular function and biological process was performed with ShinyGO 0.82 (Ge et al., 2020) and GO Ontology database (DOI:10.5281/zenodo.10536401 released 2024-01-17) for Glycine max (Aleksander et al., 2026; Ashburner et al., 2000) using the default settings (**Supplementary Table 7**).

## 3 Results

### 3.1 Excess molybdenum impairs plant growth and induces oxidative stress

#### 3.1.1 Biomass and protein concentrations

Exposure to excess molybdenum (0.5 mM Na_2_MoO_4_) led to a significant reduction (p < 0.05) in root and shoot fresh weight, as well as shoot dry weight of N_2_ fixing soybean plants (**Table 1**). However, overall plant growth, including root and shoot length, and the number and dry weight of nodules was not significantly affected by Mo addition (**Table 1**).

**Table 1:** Shoot (A) and root (B) length (cm), fresh weight (g), dry weight (mg), nodule count, nodule weight as well as total soluble protein concentrations (μg mg^-1^ FW.) of soybean (*Glycine max* cv. Primus) grown semi-hydroponically without (Control) or with molybdenum (0.5 mM Mo) and two different nitrogen supply regimes (N fix: inoculated with *B. japonicum*, week 1&2 0.25 mM KNO_3_ & week 3–5 zero N; N fed: week 1–5–10 mM KNO_3_).

		N fed				N fix			
		Control		0.5 mM Mo		Control		0.5 mM Mo	
	Unit	Means ± S.E.	sig.	Means ± SE	sig.	Means ± SE	sig.	Means ± SE	sig.
(A) SHOOT									
Length	cm	68.3 ± 6.07	A	66.25 ± 4.01	A	67.3 ± 7.27	A	60.75 ± 3.22	A
Fresh Weight	g	4.76 ± 0.11	A	5.38 ± 0.34	A	4.52 ± 0.24	A	3.23 ± 0.25	B
Dry Weight	mg	1.27 ± 0.06	A	1.34 ± 0.14	A	1.29 ± 0.09	A	0.96 ± 0.09	B
Protein Content Leaf	μg mg-1 FW	116 ± 13.1	A	84.21 ± 28.07	A	98.4 ± 4.09	A	152.92 ± 39.03	A
(B) ROOT									
Length	cm	26.8 ± 2.29	A	24.5 ± 0.87	A	28 ± 3.19	A	25.75 ± 2.1	A
Fresh Weight	g	2.97 ± 0.18	A	2.6 ± 0.37	A	2.68 ± 0.06	A	1.88 ± 0.19	B
Dry Weight	mg	0.25 ± 0.02	A	0.24 ± 0.04	A	0.32 ± 0.03	A	0.22 ± 0.02	A
Nodule Count	pcs	4.5 ± N.D.	N.D.	1 ± N.D.	N.D.	21 ± 3.49	A	18.5 ± 1.85	A
Nodule Weight	g DW	0.003 ± N.D.	N.D.	0.004 ± N.D.	N.D.	0.08 ± 0.01	A	0.07 ± 0.002	A
Protein Content Root	μg mg-1 FW	12.1 ± 3.29	A	5.05 ± 0.69	A	6.8 ± 0.29	A	7.4 ± 0.74	A

Values are means ± S.E. (biomass n = 4, protein and pigment content n = 3–4). Letters indicate significant differences between the different molybdenum treatments and nitrogen regimes (ANOVA, *post hoc* DGC, p < 0.05). For nodule weight and nodule count, statistical analysis was not performed for nitrogen fertilized-plants (N fed) due to insufficient replication (n ≤ 2); data points represent individual values or the mean of available replicates where applicable. Standard errors and significance could not be calculated and are marked as N.D. (Not determined).

**Figure 1:**
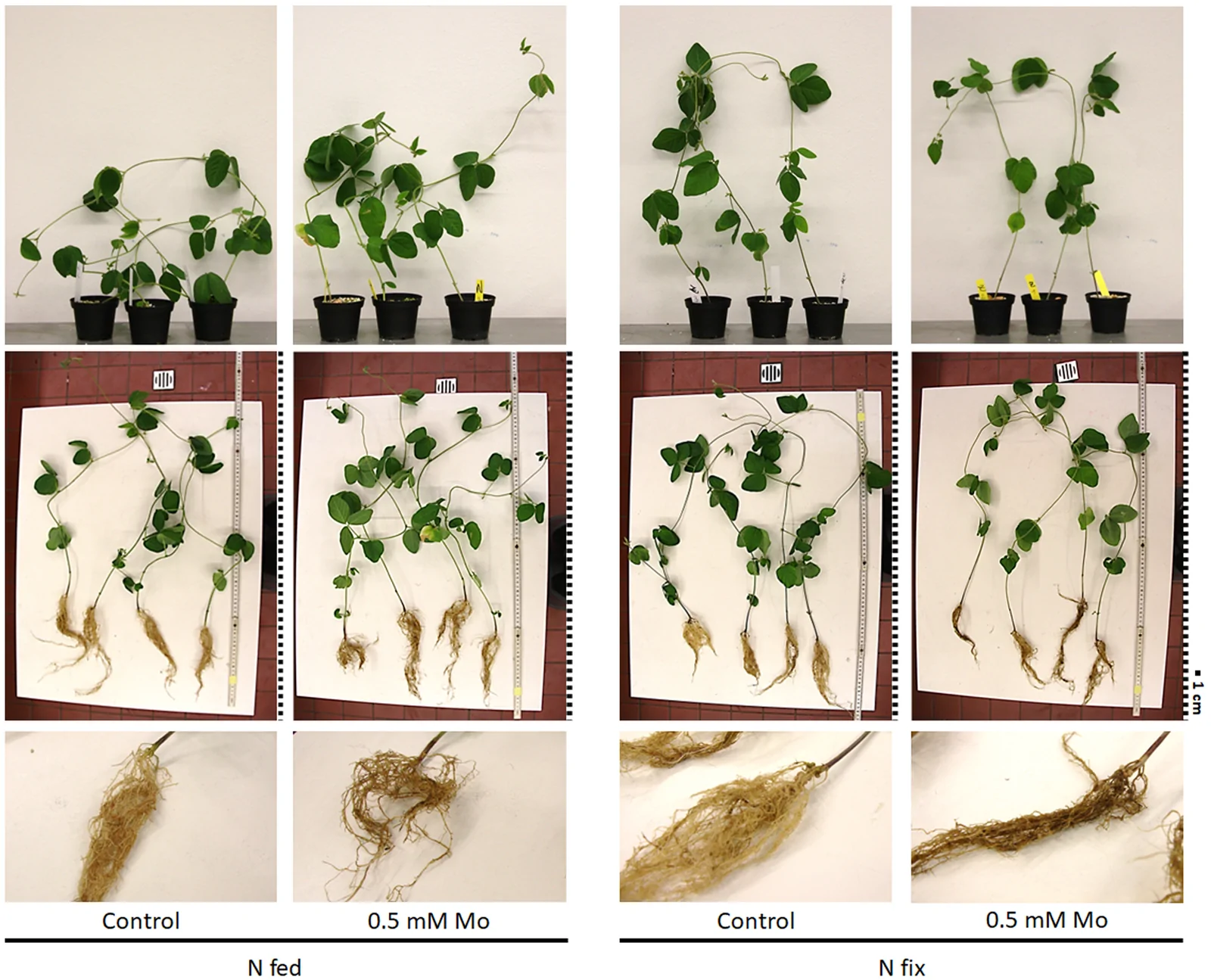
Photos of soybean plants (*Glycine max* cv. Primus) grown semi-hydroponically without (Control) or with molybdenum (0.5 mM Mo) and two different nitrogen supply regimes (N fix: inoculated with *B. japonicum*, week 1&2 0.25 mM KNO_3_ & week 3–5 zero N; N fed: week 1–5–10 mM KNO_3_). Brightness was increased by +40% in all pictures, pictures of whole plants on the table were also sharpened by +50%. For better comparability pictures were resized and a graphical representation of the scale was added. Each square represents 1 cm.

Roots of both N regimes showed clear signs of oxidative damage such as root browning and hampered lateral root development. While roots of N fix plants had a darker appearance, roots of N fed plants additionally showed signs of breakage (**Figure 1**; **Supplementary Figure 1**).

Although not statistically significant, protein content of leaves and roots was higher in the Mo N fix treatment and lower in the Mo N fed treatment compared to their respective controls (**Table 1**).

#### 3.1.2 Photosynthetic pigments, phenolic compounds and antioxidant capacity

**Figure 2:**
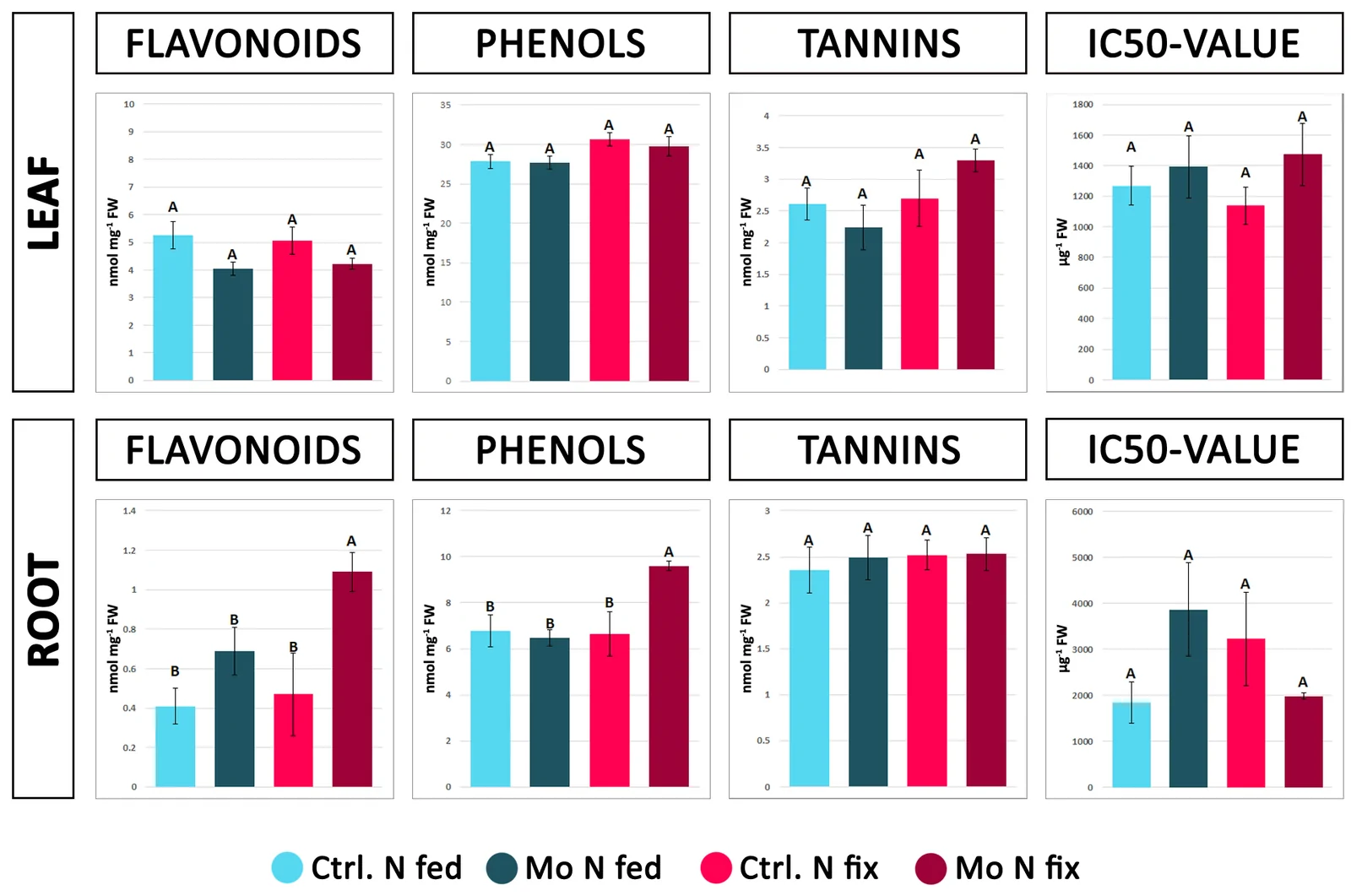
Concentrations of total flavonoids, phenolic compounds, tannins as well as IC50 values in leaf and root tissue of soybean (*Glycine max* cv. Primus) grown semi-hydroponically without (Ctrl.) or with 0.5 mM molybdenum (Mo) and two different nitrogen supply regimes (N fix: inoculated with *B. japonicum*, week 1&2 0.25 mM KNO_3_ & week 3–5 zero N; N fed: week 1–5–10 mM KNO_3_). Values are means ± S.E. (n = 3–4). Letters indicate significant differences between the different molybdenum treatments and nitrogen regimes (ANOVA, *post hoc* DGC, p < 0.05). Biomass parameters and statistics for roots and shoots are provided in **Table 1**.

Mo treatment did not cause a significant change in the concentrations of photosynthetic pigments (**Supplementary Table 1A**). Similarly, levels of total tannins, total flavonoids and total phenolic compounds remained unchanged in leaves of Mo-treated plants (**Figure 2**). Although pigment levels in Mo-treated plants were comparable to the controls, Pulse Amplitude Modulated (PAM) fluorometry revealed a significant effect on photosynthetic performance (**Supplementary Figure 2**). N fix control plants showed a significantly higher effective photochemical quantum yield (Y(II)) and relative electron transport rate (ETR), alongside significantly lower levels of non-photochemical quenching (NPQ, qN) compared to all other treatments. Mo treatment resulted in a significantly lower photochemical fluorescence quenching coefficient (qP) in N_2_-fixing plants and a significantly lower maximum photochemical quantum yield (Fv/Fm) in N fed plants (**Supplementary Figure 1**, **Supplementary Table 1B**).

In roots, levels of total flavonoids and phenolic compounds were significantly higher (p < 0.05) in Mo-treated N fix plants compared to all other treatments. This correlated with a lower IC50 value in roots of N fix plants, compared to the control and roots of N fed plants, where the IC50 value increased in response to Mo (**Figure 2**).

### 3.2 Reduced root to shoot translocation of essential nutrients in Mo-treated N fix plants

#### 3.2.1 Molybdenum concentrations

Elemental composition analysis revealed that N2 fixing control plants had significantly higher root tissue concentrations, but lower stem and leaf tissue concentrations of molybdenum compared to their non-symbiotic counterparts. In the stress treatment, however, Mo uptake and translocation were significantly higher in the N fix plants (**Table 2**).

**Table 2:** Leaf (A), stem (B) and root (C) concentrations, as well as root to shoot translocation factors (D) of macro- and micronutrients of soybean (*Glycine max* cv. Primus) grown semi-hydroponically without (Control) or with molybdenum (0.5 mM Mo) and two different nitrogen supply regimes (N fix: inoculated with *B. japonicum*, week 1&2 0.25 mM KNO3 & week 3–5 zero N; N fed: week 1–5–10 mM KNO3).

		N fed				N fix			
		Control		0.5 mM Mo		Control		0.5 mM Mo	
Organ	Unit	Means ± SE	sig.	Means ± SE	sig.	Means ± SE	sig.	Means ± SE	sig.
(A) Leaf									
Mo	mg kg^-1^	1.13 ± 1.07	C	310.75 ± 27.6	B	0.12 ± 0.06	C	720.5 ± 162.5	A
Ca	g kg^-1^	9.78 ± 0.28	A	9.57 ± 0.27	A	11.0 ± 0.58	A	8.24 ± 1.30	A
K	g kg^-1^	21.9 ± 0.76	A	23.1 ± 0.49	A	14.4 ± 0.58	B	8.40 ± 1.10	C
Mg	g kg^-1^	8.70 ± 0.22	A	8.04 ± 0.32	A	6.90 ± 0.47	A	6.04 ± 1.28	A
P	g kg^-1^	2.70 ± 0.22	A	2.36 ± 0.23	A	3.21 ± 0.27	A	2.11 ± 0.20	A
S	g kg^-1^	2.70 ± 0.17	B	2.84 ± 0.13	B	5.12 ± 0.33	A	5.70 ± 1.38	A
Fe	mg kg^-1^	99.4 ± 8.83	A	119.04 ± 26.6	A	608.04 ± 194.2	A	370.8 ± 151.9	A
Mn	mg kg^-1^	51.3 ± 2.67	B	41.6 ± 4.63	B	99.3 ± 7.96	A	43.8 ± 5.43	B
Cu	mg kg^-1^	4.63 ± 0.46	B	5.01 ± 0.66	B	6.73 ± 0.57	A	4.82 ± 0.55	B
Zn	mg kg^-1^	9.47 ± 1.23	B	10.2 ± 1.59	B	21.2 ± 1.69	A	13.7 ± 1.75	B
(B) Stem									
Mo	mg kg^-1^	1.08 ± 0.65	C	354.9 ± 17.3	B	0.39 ± 0.2	C	655.18 ± 57.2	A
Ca	g kg^-1^	3.34 ± 0.11	B	3.17 ± 0.32	B	4.66 ± 0.22	A	3.13 ± 0.16	B
K	g kg^-1^	20.35 ± 2.42	A	24.03 ± 2.5	A	12.21 ± 0.53	B	10.84 ± 0.86	B
Mg	g kg^-1^	5.57 ± 0.25	A	4.47 ± 0.17	B	5.99 ± 0.49	A	4.11 ± 0.36	B
P	g kg^-1^	1.47 ± 0.07	B	1.29 ± 0.14	B	2.21 ± 0.036	A	2.02 ± 0.10	A
S	g kg^-1^	2.49 ± 0.08	B	2.71 ± 0.04	B	4.08 ± 0.38	A	3.83± 0.44	A
Fe	mg kg^-1^	110.95 ± 7.90	B	112.73 ± 20.4	B	223.4 ± 36.3	A	255.06 ± 43.5	A
Mn	mg kg^-1^	12.69 ± 0.96	B	8.75 ± 0.55	C	24.31 ± 0.7	A	11.61 ± 0.1	B
Cu	mg kg^-1^	4.76 ± 0.41	A	4.51 ± 0.29	A	5.63 ± 0.42	A	5.79 ± 0.29	A
Zn	mg kg^-1^	5.69 ± 0.53	C	5.61 ± 0.53	C	10.07 ± 0.27	A	7.41 ± 0.38	B
(C) Root									
Mo	mg kg^-1^	0.57 ± 0.29	D	1458.7 ± 121.8	B	6.53 ± 2.2	C	2013.2 ± 99.6	A
Ca	g kg^-1^	8.69 ± 1.14	A	7.87 ± 0.64	A	5.73 ± 0.22	B	7.49 ± 0.40	A
K	g kg^-1^	36.87 ± 1.77	A	28.46 ± 1.94	B	27.1 ± 1.03	B	13.07± 1.70	C
Mg	g kg^-1^	14.80 ± 1.4	A	10.53 ± 0.98	B	6.90 ± 1.15	C	4.19 ± 0.50	C
P	g kg^-1^	2.20 ± 0.18	C	2.32 ± 0.21	C	3.35 ± 0.25	B	4.43 ± 0.22	A
S	g kg^-1^	10.50 ± 0.33	B	8.55 ± 0.71	C	12.33 ± 0.17	A	6.01 ± 0.40	D
Fe	mg kg^-1^	702.84 ± 115.65	A	497.3 ± 104.3	A	860.5 ± 319.8	A	828.5 ± 133.8	A
Mn	mg kg^-1^	73.6 ± 10.29	B	69.29 ± 11.41	B	179.6 ± 21.2	A	43.55 ± 3.93	C
Cu	mg kg^-1^	10.6 ± 0.73	B	11.12 ± 1.15	B	10.76 ± 0.63	B	15.15 ± 1.51	A
Zn	mg kg^-1^	14.8 ± 1.51	B	13.65 ± 1.75	B	25.4 ± 1.4	A	26.32 ± 2.44	A
(D) Translocation Factor (TF)									
W		4.56 ± 0.73	A	0.46 ± 0.01	B	1.13 ± 1.04	B	0.7 ± 0.13	B
Ca		1.6 ± 0.2	B	1.64 ± 0.18	B	2.72 ± 0.14	A	1.55 ± 0.08	B
K		1.15 ± 0.06	B	1.67 ± 0.13	A	0.98 ± 0.02	C	1.54 ± 0.23	A
Mg		0.97 ± 0.02	B	1.21 ± 0.13	B	1.99 ± 0.27	A	2.47 ± 0.32	A
P		1.92 ± 0.04	A	1.56 ± 0.05	B	1.63 ± 0.1	B	0.95 ± 0.04	C
S		0.49 ± 0.02	C	0.66 ± 0.06	B	0.74 ± 0.04	B	1.64 ± 0.33	A
Fe		0.33 ± 0.07	A	0.5 ± 0.08	A	1.1 ± 0.27	A	0.78 ± 0.22	A
Mn		0.94 ± 0.17	B	0.75 ± 0.09	B	0.71 ± 0.09	B	1.34 ± 0.07	A
Cu		0.88 ± 0.05	B	0.86 ± 0.04	B	1.15 ± 0.09	A	0.71 ± 0.05	C
Zn		1.03 ± 0.09	A	1.15 ± 0.04	A	1.23 ± 0.09	A	0.85 ± 0.11	A

Values are means ± SE (n = 4). Letters indicate significant differences between the different molybdenum treatments and nitrogen regimes (ANOVA, *post hoc* DGC, p < 0.05).

#### 3.2.2 Macro and micronutrients

Molybdenum treated plants showed reduced sulfate concentrations in roots of both N regimes; however, sulfate levels in leaves were not reduced (**Table 2**). Overall, N fix plants had higher sulfate levels in leaves and stems than N fed plants (**Tables 2A, B**). The presence of excess Mo also affected the levels of various other essential nutrients, especially in N fix plants. Mo-treated N fix plants exhibited lower root concentrations as well as reduced root-to-shoot translocation of potassium (K) and manganese (Mn) compared to the control and the N fed plants (**Table 2C**). Additionally, translocation of calcium (Ca), iron (Fe), phosphorus (P), copper (Cu) and zinc (Zn) was significantly (p < 0.05) reduced in these plants, which resulted in decreased concentrations of these nutrients in leaves (**Table 2D**).

### 3.3 Multivariate statistics

**Figure 3:**
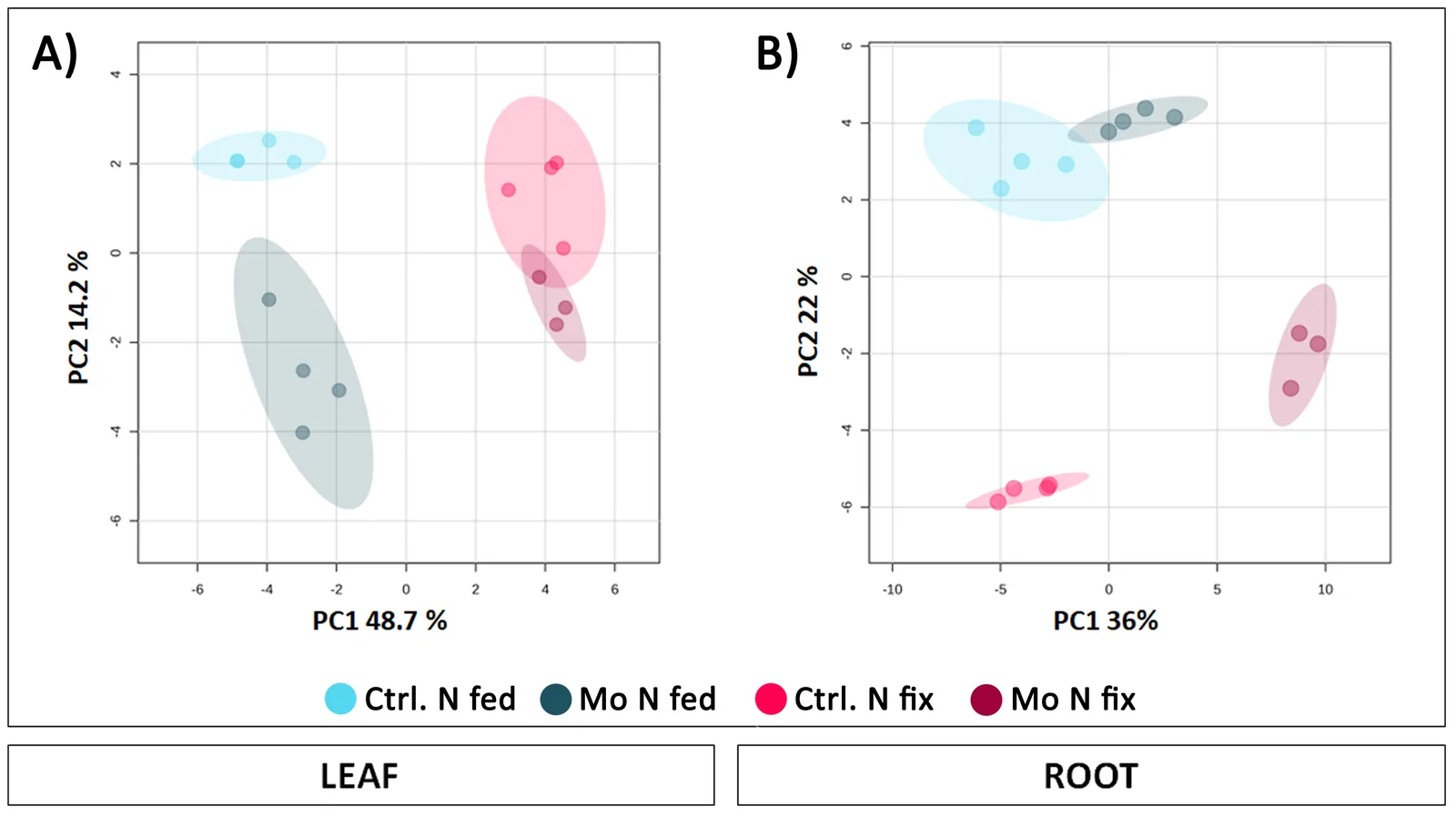
PCA plots (PC1/PC2) of leaves **(A)** and roots **(B)** including log2 transformed metabolite concentrations and LFQ intensities of significantly changed proteins (n = 3–4, ANOVA, *post hoc* Tukey, p < 0.05). Soybean plants (*Glycine max* cv. Primus) were grown semi-hydroponically without (Ctrl.) or with 0.5 mM molybdenum (Mo) in the nutrient solution and two different nitrogen supply regimes (N fix: inoculated with *B. japonicum*, week 1&2 0.25 mM KNO_3_ & week 3–5 zero N; N fed: week 1–5–10 mM KNO_3_).

The principal component analysis (PCA) showed a clear distinction between N-fertilized and symbiotically grown soybean plants (**Figure 3**). In leaves, PC1 accounted for 48.7% of the variance and separated the two N regimes. PC2 (14.2%) separated the N fed control and N fed Mo treatment (**Figure 3A**), while PC3 (11.1%) separated the N fix Mo treatment from the controls. In roots, 36% of the variance was explained by PC1, which separated the controls from the Mo treatments. PC2, which accounted for another 22%, distinguished the two N regimes (**Figure 3B**).

To identify the proteins and metabolites responsible for the separation, a threshold with the largest loadings was set at ±0.065 (**Supplementary Figure 3**). Detailed statistical results for metabolites and proteins together with their respective PCA loadings can be found in the supplements (metabolites **Supplementary Tables 2**, **S3**, proteins **Supplementary Tables 4**, **S5**).

#### 3.3.1 Leaf metabolites

**Figure 4:**
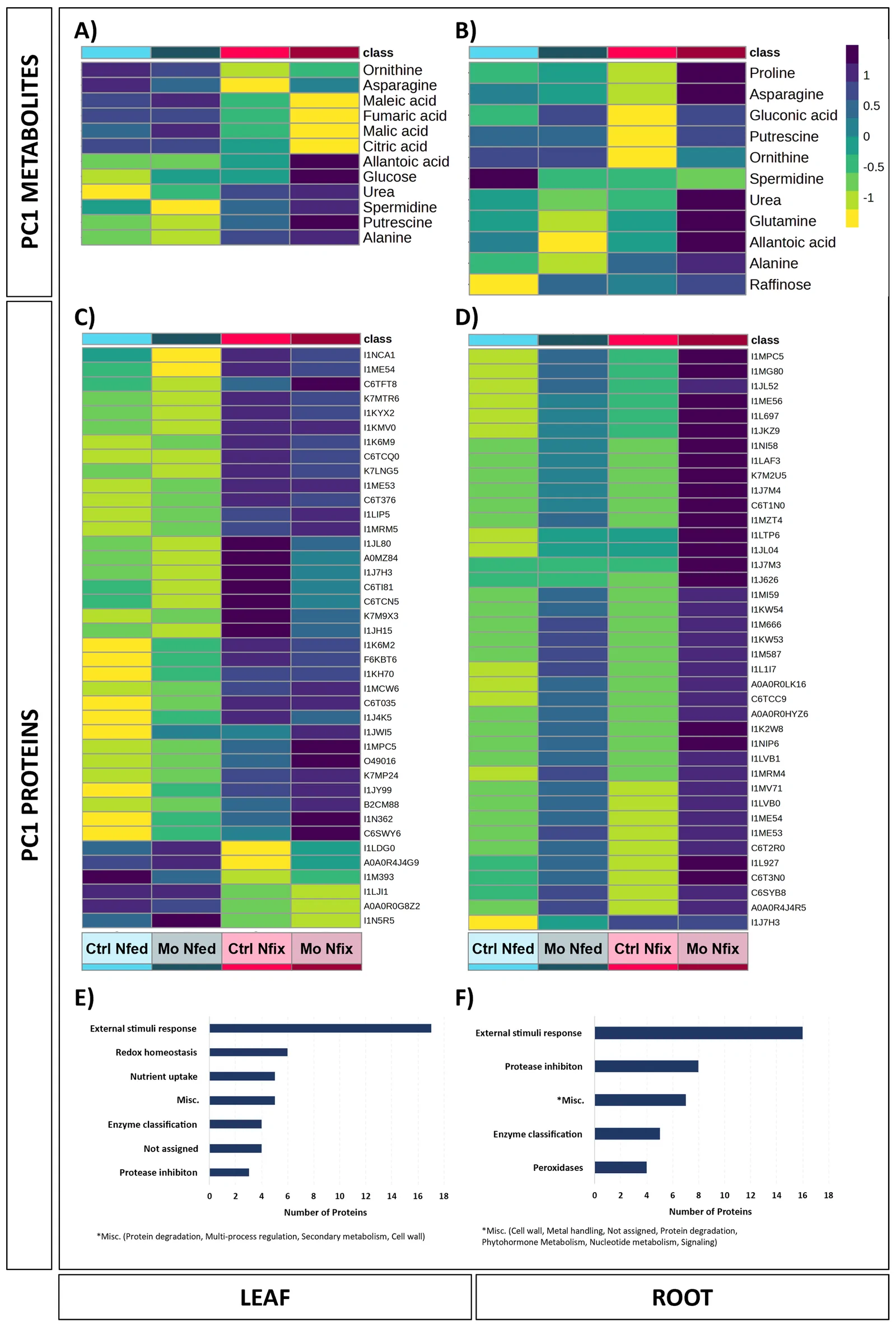
Heatmap of leaf **(A)** and root **(B)** metabolites, leaf **(C)** and root **(D)** proteins with PC1 loadings higher than ±0.65. **(E, F)** show the major functional categories of leaf and root proteins respectively with PC1 loadings higher than ±0.65. Soybean plants (*Glycine max* cv. Primus) were grown semi-hydroponically without (Ctrl.) or with 0.5 mM molybdenum (Mo) in the nutrient solution and two different nitrogen supply regimes (N fix: inoculated with *B. japonicum*, week 1&2 0.25 mM KNO_3_ & week 3–5 zero N; N fed: week 1–5–10 mM KNO_3_). Colors represent z-transformed mean values (n = 3–4). Letters indicate significant differences between the different molybdenum treatments and nitrogen regimes (ANOVA, *post hoc* Tukey, p < 0.05).

The difference between the different N regimes (PC1) was driven by changes in levels of organic acids (citric acid, fumaric acid, malic acid, maleic acid), which were more abundant in N fed plants, as well as amino acids (asparagine, ornithine, alanine), polyamines (spermidine, putrescine), nitrogen compounds (urea, allantoic acid), which were constitutively more abundant in N fix plants irrespective of Mo addition (**Figure 4A**). This difference was accentuated in the presence of Mo, as organic acids were significantly reduced, while amino acids as well as N-compounds and glucose significantly increased in the N fix Mo treatment compared to the N fix control. In the N fed plants, only asparagine was significantly reduced (**Figure 4A**).

PC2 showed that Mo treatment led to a different response in the two N regimes. While levels of urea, proline and fructose were higher in all Mo-treated plants compared to the controls, Mo treatment led to a significant decrease in amino acids (tyrosine and asparagine) as well as an increase in maltose level in leaves of N fed plants. In contrast, leaves of Mo-treated N fix plants showed a significant increase of several carbohydrates (glucose, raffinose, maltose and trehalose), asparagine, putrescine and allantoic acid. Finally, spermidine and lactic acid showed opposite trends, decreasing in N fed but increasing in leaves of N fix plants treated with molybdenum (**Supplementary Figure 3A**).

#### 3.3.2 Root metabolites

In roots, the difference between controls and Mo treatments (PC1) was determined by changes in levels of amino acids (alanine, asparagine, glutamine, ornithine and proline), polyamines (putrescine and spermidine), nitrogen compounds (allantoic acid, urea) as well as raffinose and gluconic acid. These changes were more pronounced in roots of N_2_ fixing plants (N fix), where several of these metabolites significantly increased in abundance (**Figure 4B**).

PC2, which shows the difference between the two N regimes, revealed that concentrations of several organic acids (2-oxoglutaric acid, citric acid, ascorbic acid and gluconic acid) were constitutively higher in N fed plants. In contrast, N fix controls had higher levels of amino acids (glutamine and alanine), urea, raffinose and malonic acid (**Supplementary Figure 4B**).

#### 3.3.3 Leaf proteome

Of 2,515 identified proteins in leaves, 1,668 were detected in at least half of the replicates of a given treatment. Altogether, 122 proteins showed a significantly changed abundance in response to Mo (**Supplementary Figure 5A**). This response was highly dependent on N regime, as 81 were exclusively changed in leaves of N fed plants, 39 in N fix leaves, and only two proteins were significantly changed in both N regimes (**Supplementary Figure 5A**).

PC1 (48.7%) separated the two N regimes. Proteins with the highest loadings are shown in **Figure 4C**. Proteins with the highest loadings on PC1 (**Figure 4C**) belong to the functional categories “external stimuli response” (A0A0R4J4G9, C6SWY6, C6T035, C6T376, C6T3A2, I1JCB6, I1K6M2, I1L0V9, I1ME53, I1MPC5, K7MP24, Q43453, K7M9X3, I1ME54, I1KMV0, I1JWI5, I1MRM5), “redox homeostasis” (A0A0R4J4T3, C6SZ56, I1JY99, I1M393, I1N362, K7MTR6), “nutrient uptake” (C6TCN5, I1J7H3, C6TI81, A0MZ84, I1JL80), “enzyme classification” (I1N5R5, I1KH70, I1MCW6, O49016), “protease inhibition” (A0A0R0G8Z2, I1J4K5, I1KYX2) (**Figure 4E**). Functional categories with fewer than two proteins were grouped together under the term miscellaneous (*Misc.: Protein degradation, Multi-process regulation, Secondary metabolism, Cell wall). Of the proteins with the highest loadings on PC1, non-heme ferritins (C6TCN5, I1J7H3, C6TI81, A0MZ84, I1JL80), a matrixin-type metalloprotease (I1JH15) and a SCP domain-containing protein (K7M9X3) showed a significantly lower abundance, while a peroxidase (I1N362) showed a significantly higher abundance in Mo-treated N fix leaves, compared to the N fix control (**Figure 4C**).

#### 3.3.4 Root proteome

Of the 3,518 proteins identified in roots, 2,058 were detected in at least two of four replicates. Molybdenum treatment induced significant changes in the abundance of 357 proteins. The majority (63.3%) of these proteins were exclusively affected in N fix plants (**Supplementary Figure 5B**). In contrast, only 18.5% were changed exclusively in N fed roots, and another 18.2% were common to all Mo-treated plants (**Supplementary Figure 5B**). In both treatments, approximately 57% of the significantly changed proteins were decreased and 43% increased in abundance.

The principal component analysis showed a clear separation between the controls and Mo treatments on PC1 (**Figure 3**). However, this separation was more pronounced in the N_2_ fixing plants. Proteins contributing most to the difference between control and Mo-treated roots can be assigned to the functional categories “external stimuli response” (C6T376, I1K6M2, A0A0R0LK16, C6T1N0, C6T2R0, C6T397, C6T3N0, I1JCB6, I1L927, I1LEI5, I1M587, I1ME53, I1ME54, I1ME56, I1MPC5, I1MRM4), “protease inhibition” (C6TCC9, I1KW53, I1KW54, I1LVB0, I1LVB1, I1MI59, I1NI58, B1ACD2), “enzyme classification” (A0A0R4J4R5, I1K2W8, I1MG80, I1NAC8, C6T376, I1K6M2) as well as “peroxidases” (I1J7M3, I1J7M4, I1JKZ9, I1MZT4). The functional category miscellaneous (cell wall, metal handling, not assigned, protein degradation, phytohormone metabolism, nucleotide metabolism, signaling) comprises categories with fewer than two proteins (**Figures 4D, F**).

#### 3.3.5 Comparison to tungsten stress response

##### 3.3.5.1 Leaves

**Figure 5:**
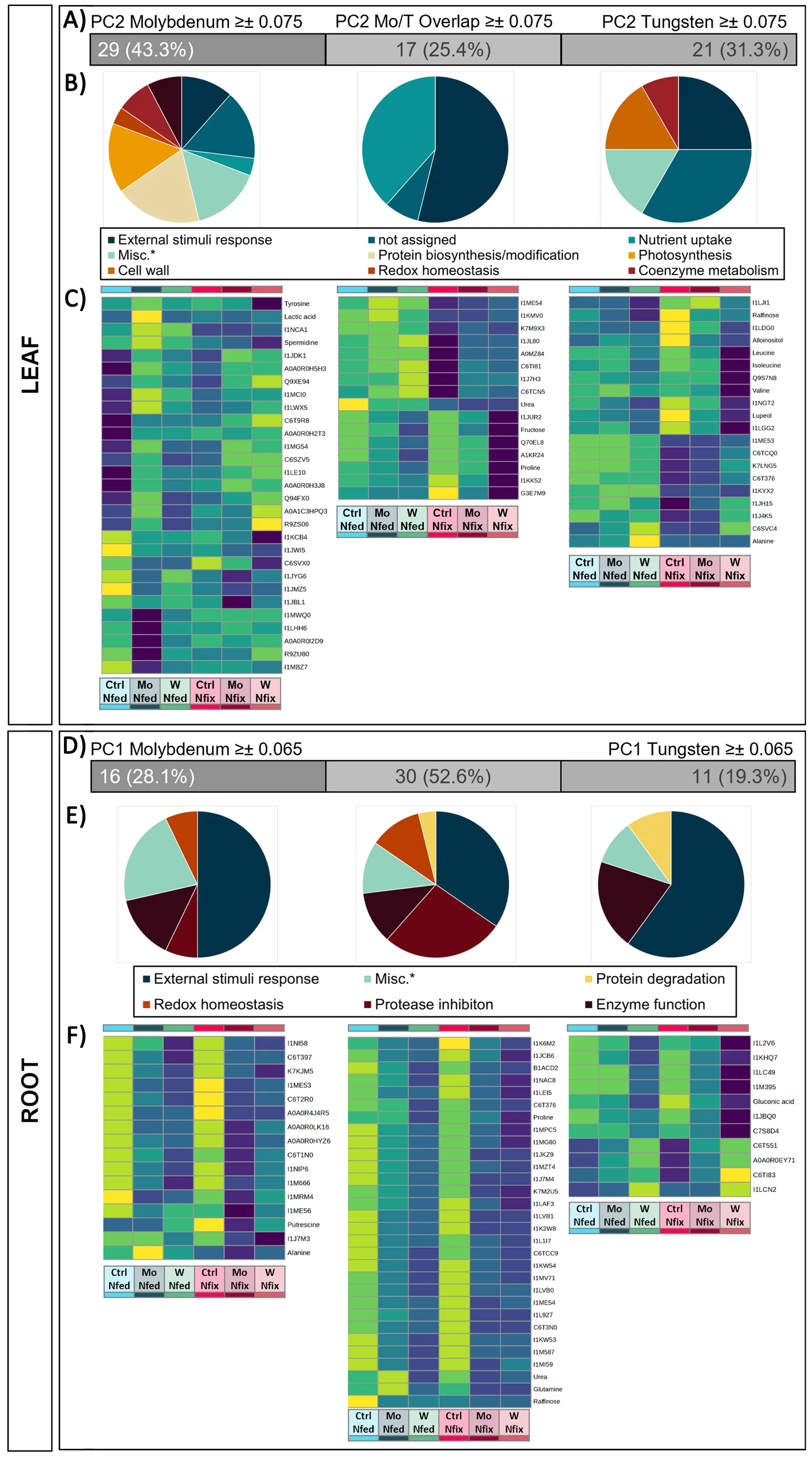
Comparison of significantly changed proteins and metabolites that contribute most to PC2 in leaves and PC1 in roots and thus to the difference between controls and treatments during W and Mo treatments. Numbers and percentages of metabolites and proteins of leaves **(A)** with PC2 loadings higher than ±0.075 that showed significantly changed abundances in Mo-treated plants, in both Mo- and W-treated plants as well as only in W-treated plants. Pie charts show functional categories of leaf proteins significantly changed in Mo, W or both treatments **(B)**. Heatmaps of significantly changed metabolites and proteins (ANOVA, *post hoc* Tukey, p < 0.05) in leaves with PC2 loadings higher than ±0.075 **(C)**. Numbers and percentages of metabolites and proteins of roots **(D)** with PC1 loadings higher than ±0.065 that showed significantly changed abundances in Mo-treated plants, in both Mo- and W-treated plants as well as only in W-treated plants. Pie charts show functional categories of root proteins with PC1 loadings higher than ±0.065 significantly changed in Mo, W or both treatments **(E)**. Heatmaps of significantly changed metabolites and proteins (ANOVA, *post hoc* Tukey, p < 0.05) in roots with PC1 loadings higher than ±0.065 **(F)**. Soybean plants (*Glycine max* cv. Primus) were grown semi-hydroponically without (Ctrl.) or with 0.5 mM molybdenum (Mo) or tungsten (W) in the nutrient solution and two different nitrogen supply regimes (N fix: inoculated with *B. japonicum*, week 1&2 0.25 mM KNO_3_ & week 3–5 zero N; N fed: week 1–5–10 mM KNO_3_). Colors represent z-transformed mean values (n = 3–4).

Combining the highest loadings (± 0.075) on the principal components responsible for the separation between controls and molybdenum-treated plants (PC2), as well as controls and tungsten-treated plants resulted in a set of 67 significantly changed proteins and metabolites. Of these, 17 (25.4%) proteins and metabolites were shared between tungsten and molybdenum treatment, 29 (43.3%) were specific to molybdenum-treated plants and 21 (31.3%) were tungsten-specific (**Figure 5A**; **Supplementary Table 6**).

The heatmaps in **Figure 5C** show the abundance patterns of these leaf proteins and metabolites across all treatments and N-regimes. The molybdenum-specific response was driven primarily by proteins of the functional categories photosynthesis and coenzyme metabolism, which were significantly reduced in abundance in Mo- and W-treated plants compared to the controls (**Figure 5C**). Another set, contributing to the Mo-specific response, consisted of proteins with increased abundance in the Mo- and W- treated plants (external stimuli response, protein modification/biosynthesis), as well as tyrosine, spermidine and lactic acid, which were decreased in leaves of Mo-treated N fed plants and increased in N fix plants (**Figure 5C**; **Supplementary Tables 6A, B**). One set of proteins belonging to the functional categories enzyme classification, protein biosynthesis was exclusively more abundant in Mo-treated N fed plants compared to all other treatments (**Figure 5B**; **Supplementary Table 6A**).

The shared response between Mo- and W-treated plants was dominated by proteins of the functional category external stimuli response as well as nutrient uptake which were significantly more affected in the N fix treatments compared to the control (**Figures 5B, C**). While proteins of the functional category external stimuli response mostly increased in abundance (Pathogenesis-related protein 1, Dehydrin, Annexin, BURP domain-containing protein), abundance of proteins of the category nutrient uptake decreased (non-heme ferritins) (**Figures 5B, C**; **Supplementary Table 6A**). Additionally glucose, fructose, proline and urea were significantly increased in both Mo- and W-stressed N fix leaves, as well as leaves of W-stressed N fed plants (except for urea) (**Figure 5C**; **Supplementary Table 6B**).

The tungsten specific response is primarily mediated by proteins of the category external stimuli response, not assigned, cell wall, as well as metabolites significantly affected in W-treated N fix plants (**Figure 5B**; **Supplementary Tables 6A, B**).

GO enrichment analysis revealed that proteins involved in iron transport, cellular iron-ion homeostasis and sequestration (GO:0006826, GO:0006875, GO:0006879, GO:0006880), flower development (GO:0009908), response to hormone and endogenous stimulus (GO:0009725, GO:0009719) as well as response to biotic stimulus (GO:0009607) and organic substance (GO:0010033) were significantly enriched terms in both treatments (**Supplementary Figure 6**; **Supplementary Table 7**).

##### 3.3.5.2 Roots

Combining the significantly changed proteins and metabolites with the highest loadings on PC1 (± 0.065) revealed that a majority (52.6%) of proteins and metabolites were shared among the Mo and W treatments (**Figure 5D**; **Supplementary Table 6**). These included proline, urea and glutamine, together with proteins belonging to the functional categories external stimuli response, protease inhibition, enzyme function and redox homeostasis, which showed significantly increased abundances in Mo- and W- treated plants of both N-regimes relative to the respective controls, although the effect was more pronounced in the N fix plants for a number of these proteins and metabolites (**Figures 5E, F**; **Supplementary Tables 6C, D**). Among the proteins and metabolites specific to tungsten-treated plants (19.3%), the functional categories external stimuli response, enzyme function and protein degradation, together with gluconic acid, drove the tungsten-specific response (**Figures 5E, F**). In Mo-treated plants (28.1%), by contrast, putrescine, alanine and proteins of the categories external stimuli response, enzyme function and miscellaneous (metal handling, nucleotide metabolism, not assigned) drove the response (**Figures 5E, F**).

The heatmaps show that the response in N fix plants was more pronounced than in N fed plants, while following the same pattern, indicating a shared response that differed in magnitude rather than direction between the two N-regimes (**Figure 5F**). Moreover, with exception of the Mo specific response, abundances scaled progressively from control to Mo to W, consistent with a shared but increasingly severe response across the two metals (**Figure 5F**).

The GO enrichment analysis revealed that especially processes related to the negative regulation of catalytic activity, proteolysis and molecular function (GO:0051346, GO:0045861, GO:0010951, GO:0010466, GO:0006952, GO:0051336, GO:0043086, GO:0044092) were significantly enriched GO terms (**Supplementary Figure 6**; **Supplementary Table 7**).

## 4 Discussion

This study investigates the metabolic response of symbiotic (N fix) and KNO_3_-fertilized (N fed) soybean plants (*Glycine max*) exposed to excess concentrations of the essential plant micronutrient Mo and compares it to the stress response under exposure to its chemical analog tungsten (W).

Our results demonstrate that symbiotically grown plants exhibited higher sensitivity to excess Mo compared to nitrogen-fertilized plants, possibly due to a greater Mo demand leading to continuous uptake even under toxic levels. In addition, we found that excess Mo impaired photosynthetic efficiency, caused oxidative stress and disrupted nutrient translocation to the shoots, particularly for essential nutrients and cofactors such as Fe, Cu, Mn and S. Furthermore, impaired biosynthetic sinks and reduced growth resulted in a disruption of nitrogen metabolism, which led to an accumulation of ureides and amino acids as well as protective metabolites. While the quality of the observed stress response matches the W-induced stress response (Preiner et al., 2024), the intensity as well as the metabolic consequences and stress severity differed between the two transition metals. Throughout this discussion, stress severity and sensitivity to the transition metals are inferred from a combination of biomass parameters (root and shoot dry weight, root and shoot length), physiological indicators such as photosynthetic efficiency, antioxidative capacity (IC50), as well as the magnitude of the protective metabolite accumulation (flavonoids, phenolics, polyamines, proline and gluconic acid) (**Supplementary Tables 8A–C**).

### 4.1 N-regime determines sensitivity to excess Mo

Our results demonstrate that the tolerance to excess molybdenum is dependent on the mode of nitrogen nutrition, as N_2_-fixing plants (N fix) responded more strongly to excess Mo in the nutrient solution. While roots of both N-regimes showed visible symptoms of metal toxicity, such as browning and hampered lateral root development, the biomass and physiological parameters in the Mo-treated N-fertilized plants (N fed) remained comparable to the control (**Figure 1**). In contrast, symbiotically N_2_-fixing plants (N fix) showed a significant reduction of root and shoot fresh weight as well as root dry weight upon exposure to excess Mo (**Table 1**). This differential sensitivity of the two N-regimes seems counterintuitive, as symbiotically grown legumes are known to have a higher demand for Mo than plants grown without N_2_-fixing symbionts (Bursakov et al., 2023; Parker and Harris, 1977). However, this higher nutritional demand might paradoxically make N fix plants more sensitive to excess Mo. As N fix plants accumulated significantly more molybdenum (**Table 2**) than their N fed counterparts, it seems plausible that this higher nutritional requirement in N_2_-fixing soybeans drives continuous Mo uptake, even if external concentrations exceed physiologically optimal levels. While association with symbiotic nitrogen-fixing bacteria provides significant benefits in inducing resilience to several abiotic and biotic stresses (Larrainzar et al., 2007; Staudinger et al., 2012, 2016; Turetschek et al., 2019), the nutritional demand of symbiosis can also limit plant growth, and in the case of excess Mo, lead to inefficient nutrient uptake and translocation. Future research may focus on understanding this nutrient translocation imbalance observed in symbiotic plants (N fix) and the role that symbiotic nitrogen fixation plays in this respect.

### 4.2 Excess Mo impairs photosynthesis and disrupts nutrient homeostasis in N fix plants

Although photosynthetic pigment contents were not affected (**Supplementary Table 1A**), Mo-treated N fix plants showed a lower effective photochemical quantum yield (Y(II)), ETR (Relative electron transport rate), as well as significantly higher levels of non-photochemical quenching (NPQ) compared to the control (**Supplementary Figure 2**; **Supplementary Table 1B**), indicating impaired photosynthetic efficiency under excess Mo. This is consistent with the well-documented sensitivity of the photosynthetic apparatus to transition metal stress (DalCorso et al., 2013), and potentially also serves as an explanation for the growth reduction (**Table 1**). In line with this observation, excess Mo resulted in a disruption of the homeostasis of several essential nutrients, as the root-to-shoot translocation of S, K, Mn, Ca, P, Cu, Fe and Zn was significantly altered in N fix plants (**Table 2D**). This systemic nutrient imbalance likely contributed to the observed hampered growth, reduced photosynthetic efficiency and oxidative stress in N fix plants (**Figures 1**, **2**; **Supplementary Table 1B**), as several of the affected elements such as Ca, Mn, Fe and Cu are essential cofactors in photosynthetic enzymes and antioxidative enzyme systems (DalCorso et al., 2013).

Furthermore, our study showed a particular interference of excess Mo with the uptake of sulfate, which is another essential nutrient for plant growth. While the observed effect on sulfur concentrations was again not as pronounced in N fed plants, root sulfate concentrations of N fix plants dropped by approximately half compared to the control (**Table 2C**), which potentially also impaired plant growth. This, however, did not affect root-to-shoot translocation, as there was no reduction of S content in shoots and leaves of the Mo-treated plants (**Tables 2A, B**). This pattern is consistent with both the observed altered root morphology, and a competitive inhibition of sulfate uptake by molybdate at the root level due to their chemical and structural similarity. Fitzpatrick et al. (2008) showed that high free MoO_2–_^4^ concentrations above 34 μM competitively inhibited uptake of SO_2–_^4^ by the plant sulfate transporter SHST1 from *Stylosanthes hamata* expressed in yeast, which represents a plausible molecular basis for the reduced root sulfate concentrations observed. Given the central role of sulfur as part of Fe-S clusters of both the Fe protein and the FeMo-cofactor of nitrogenase, another potential explanation for the reduced root sulfur concentrations might be a higher S demand in nodules due to enhanced nitrogenase enzyme synthesis (Schneider et al., 2019). However, a direct effect on nitrogenase enzyme levels and activity remains to be tested.

In this regard, the effects on Fe homeostasis are also notable. N fix plants showed not only a reduced root-to-shoot translocation of Fe (**Table 2D**) but also a significant decrease in ferritins in leaves and roots (**Supplementary Figure 7**), which are the primary iron storage proteins in plants, binding free iron and buffering excess Fe during xylem unloading (Briat et al., 2010; Printz et al., 2016). The decrease in ferritin abundances in turn might have contributed to increased oxidative stress in N fix plants (Ravet et al., 2009). A functional relationship between iron and molybdenum metabolism has been previously reported in cucumber, where Fe-deficient conditions led to significantly increased root Mo concentrations and reduced leaf tissue concentrations, indicating an interdependent regulation of these two elements (Vigani et al., 2017). As most plant molybdoenzymes also contain iron-containing redox groups, an imbalance in either metal affects central enzymes of various important metabolic pathways (Vigani et al., 2017). One possible explanation for the more severe effect on N fix plants is that this interplay between Mo and Fe was further affected by the Fe demand of the bacterial nitrogenase enzyme complex itself, as it contains both iron-molybdenum and iron-sulfur clusters (Burgess, 1990; Burgess and Lowe, 1996). Excess Mo might have stimulated nitrogenase enzyme production, thereby increasing Fe demand in nodules and roots and reducing its translocation to aerial parts, consequently hampering metabolic processes and growth. Although not directly tested in this study, it would also explain why ferritin abundance was lower in roots in the excess Mo treatment, as iron is primarily shuttled to the nodules for nitrogenase and leghemoglobin synthesis (Chikoti et al., 2020; Ragland and Theil, 1993). These patterns illustrate that excess Mo acted primarily at the root level through impaired root-to-shoot translocation of essential nutrients (i.e., S, K, Mn, Ca, P, Cu, Fe and Zn), and that the leaf-level effects, discussed below, may largely be a consequence of the perturbations in the roots.

Although photosynthetic efficiency was impaired in the excess Mo condition, we observed a significant increase in certain soluble carbohydrates (glucose, raffinose, sucrose) and a reduction of starch concentrations in leaves of Mo-treated N fix plants (**Supplementary Table 2A**). When photosynthetic activity is reduced by environmental constraints, plants are known to accumulate sugars and break down starch to ensure sufficient supply to cellular respiration and maintain osmotic balance (Thalmann and Santelia, 2017). However, apart from enhanced resource allocation to meet the plant’s as well as the symbionts’ higher energy demand during stress, the observed accumulation of soluble sugars together with a reduction of carbohydrate storage pools might also be interpreted as a symptom of reduced turnover as well as stalled growth. Together with the greater metabolic investment required to maintain the resource-intensive rhizobia symbiosis under stress, this likely contributed to the observed reduced biomass production, metabolic perturbations and relatively higher stress severity in the N fix treatment compared to N fed plants.

### 4.3 Excess Mo induces systemic accumulation of protective metabolites in symbiotic plants

The principal component analysis revealed that the main driver of variation differed between the two organs (**Figures 3A, B**). In leaves, PC1 (48.7%) separated the two nitrogen regimes, while PC2 (14.2%) separated the molybdenum treatments from the controls (**Figure 3A**). In roots, on the other hand, PC1 (36%) separated controls from Mo-treated plants, with the N-regime effect separated on PC2 (22%) (**Figure 3B**). This indicates that the effect of the different N-regimes on leaf metabolic processes was stronger than the effect of excess Mo, whereas root metabolic processes were dominated by the direct response to molybdenum. The lower proportion of variance explained by PC1 in roots is consistent with this, as the variation in roots was distributed across a second strong axis showing the N-regime effect (PC2). These patterns are consistent with roots being the primary site of Mo uptake, chelation and sequestration, while leaves integrate whole-plant nitrogen status and respond to excess Mo largely indirectly, through altered nutrient supply from the root.

Consistent with roots being the primary site of Mo response, physical symptoms such as root browning and hampered lateral root development (**Figure 1**) indicate that molybdenum causes oxidative stress at the root level if present in excess concentrations. This is further supported by the observed significant accumulation of various well-established protective compounds such as flavonoids, phenolic compounds, putrescine and proline (**Figures 2**, **4A, B**) in N fix plants, where the highest Mo concentrations were measured (**Table 2**). This accumulation of flavonoids and phenolic compounds in roots of N fix plants was accompanied by a higher, though not significant, *in vitro* antioxidant capacity (lower IC50 value), indicating a local antioxidative response (**Figure 2**). This is consistent with the well-documented role of phenolic compounds as reactive oxygen species (ROS) scavengers under abiotic stress (Haider et al., 2026; Sytar et al., 2013). The observed higher levels of flavonoids and phenolic compounds in roots of N fix plants, however, also highlight their dual role in metal chelation and sequestration. Flavonoids are known to be involved in Mo complexation and potentially sequestration to vacuoles in *Brassica juncea*, forming a water-soluble blue complex (Hale et al., 2001). This dual metabolic function during excess Mo exposure also explains the strong induction of flavonoids in root tissue of N fix plants.

The notion that Mo, similar to other transition metals, causes oxidative stress is not a novel finding. Xu et al. (2018) found increased levels of antioxidative enzymes such as catalase (CAT), peroxidase (POD), ascorbate peroxidase (APX), and flavonoids in roots, along with CAT and superoxide dismutase (SOD) in leaves of soybean seedlings grown under excess Mo conditions (100 mg L^-1^). In our experiment, we found certain peroxidases to be increased in abundance in Mo-treated roots (**Supplementary Table 5**), suggesting that the observed higher antioxidative capacity found in N fix plants is primarily attributable to the higher levels of non-enzymatic protective compounds rather than a broad enzymatic shift. One reason for this might be a lower stress severity at the comparatively lower molybdenum concentration of 0.5 mM Mo (48 mg L^-1^) applied in our experiment, as well as the fact that Xu et al. (2018) used soybean seedlings in their experiment.

Apart from flavonoids and phenolic compounds, principal component analysis (PCA) revealed a broader role of various known stress-associated metabolites such as proline and polyamines (putrescine) as well as their precursors glutamate and ornithine in the plant’s response to excess Mo (**Figure 4**). Polyamines (PAs) are thought to play an important role in stabilizing membrane systems, protecting nucleic acids and cellular processes from induced oxidative damage by modulating enzyme activity and plant developmental processes during heavy metal (HM) stress (Groppa et al., 2007; Menéndez et al., 2019; Sharma and Dietz, 2006), while proline is thought to contribute to osmotic adjustment, ROS scavenging and metal chelation, as well as stabilizing proteins and membranes (Kumchai et al., 2013; Spormann et al., 2023; Szabados and Savouré, 2010). The metabolic plasticity of symbiotic plants in our experiment is further underlined by gluconic acid, another protective compound, which also contributed to the separation between controls and the excess Mo treatments in roots (PC1). Although its increase in abundance was not significant, this may reflect the importance of chelating excess Mo, as gluconic acid has previously been found to reduce Mo toxicity by forming stable complexes and restricting root-to-shoot translocation (Ramos et al., 1997; Spence and Kallos, 1963; Xu et al., 2018). However, at levels of gluconic acid observed in this experiment, Mo translocation was not significantly reduced relative to controls (**Table 2D**). Furthermore, it has to be noted that the observed induction of protective compounds (i.e. flavonoids, phenolic compounds, putrescine and proline) did not translate into reduced stress severity (i.e. improved photosynthesis or restored fresh/dry weight) (**Table 1**; **Supplementary Table 1**). In addition, the antioxidative and chelation response discussed above was mostly localized to the roots, consistent with their role as the primary site of Mo accumulation (**Table 2**), whereas changes in leaf metabolite levels reflected disturbed carbon and nitrogen metabolic processes.

### 4.4 Excess Mo disrupts nitrogen metabolism in N fix plants

Excess Mo led to a variety of perturbations in nitrogen metabolism, particularly in N fix plants. Apart from the protective N-containing compounds, PCA also showed a significant accumulation of nitrogen metabolic intermediaries such as allantoic acid, glutamine, asparagine and urea (**Figures 4A, B**). In contrast to the root-localized antioxidative response, this accumulation of nitrogenous compounds occurred in both roots and leaves, reflecting the systemic nature of the nitrogen-metabolic disruption. Under normal conditions, bacteroid-derived ammonia is rapidly assimilated into glutamine and glutamate via the glutamine synthetase/glutamine-oxoglutarate aminotransferase (GS/GOGAT) pathway and then channeled into ureide synthesis for export to sink tissues. However, the observed accumulation of glutamine and glutamate in roots as well as ureides and nitrogenous compounds in roots and shoots (**Figures 4A,B**), together with stable concentrations of GS/GOGAT enzyme abundances (**Supplementary Tables 4**, **S5**) indicates that under Mo excess conditions, biosynthetic sinks cannot match the rate at which assimilated nitrogen is exported from the nodules. Although we did not directly measure ammonia, this might have resulted in an ammonia accumulation in the cytosol of nodules, which itself can cause toxicity and potentially lead to feedback inhibition of N_2_ fixation. Additionally, PCA revealed a role of protease and peptidase inhibitory proteins in Mo stress response (**Figures 4E, F**). Together with stable or slightly increased soluble protein concentrations in leaves and roots of N fix plants (**Table 1**), this indicates an active suppression of proteolysis as an additional contributor to increased ammonium levels inside the cells (Paschalidis et al., 2019). Thus, the concomitant accumulation of polyamines as well as free amino acids such as asparagine and proline could not only be seen as an active protective mechanism, but also as a means to transiently sequester excess nitrogen to prevent ammonia accumulation, and balance C/N levels (Balestrasse et al., 2005; Paschalidis et al., 2019). Taken together, we hypothesize that under excess Mo conditions, impaired biosynthetic sinks for assimilated nitrogen disrupted nitrogen metabolism by causing an accumulation of ureides and amino acids and potentially resulting in feedback inhibition of N_2_ fixation. Although our data do not directly show this, various studies have reported that excess Mo results in an inhibition of N_2_ fixation, a reduction of nodule mass and number as well as structural changes in nodule tissue (Alam et al., 2015; Yang et al., 2020). In contrast to N fix plants, N fed plants showed no significant changes in growth or N-metabolic intermediaries (**Table 1**, **Supplementary Table 2**, **S3**) under excess Mo, and nitrogen-assimilatory enzyme abundances, apart from glutamine synthetase, showed no consistent significant change (**Table 1**; **Supplementary Tables 4**, **S5**). This is consistent with findings of Kevresan et al. (2001), who demonstrated that excess Mo led to an increase in nitrate reductase (NR) activity and a decrease in glutamine synthetase (GS) activity. To conclusively verify this hypothesis and to establish a direct link between the accumulation of N compounds and inhibition of N_2_ fixation during transition element stress, the direct effect of Mo on N_2_ fixation and on nitrogen export at the nodule-symbiont interface as well as nitrate assimilation remains to be resolved.

### 4.5 Molybdenum and tungsten elicit a qualitatively similar but quantitatively distinct metabolic stress response

Our results show that, while molybdenum elicits a similar metabolic response to that previously reported for tungsten (Preiner et al., 2024), both the stress severity and the magnitude of the observed response were lower for Mo than for W (**Supplementary Table 8A**). Furthermore, we found that, similarly to W stress (Preiner et al., 2024), N fix plants mounted a stronger metabolic response than N fed plants under excess Mo conditions (**Supplementary Table 8C**). This suggests that the symbiotic association is a key driver of the observed metabolic plasticity under both Mo and W stress. However, as discussed above, this symbiotically enhanced production of protective compounds did not translate into an improved tolerance or restored biomass production (fresh weight/dry weight) in any stress treatment.

In summary, shared features of Mo and W stress included a disruption of Fe-buffering systems by ferritins and a comparable pattern of starch depletion together with a soluble carbohydrate accumulation (**Supplementary Figure 6**; **Supplementary Tables 2**, **S3**). Beyond primary metabolism, we also observed a co-induction of various external stimuli response proteins, together with protective metabolites such as flavonoids, polyamines and proline in both Mo- and W-treated soybeans across both plant organs, although of considerably higher magnitude under W (**Figure 5**; **Supplementary Tables 6**, **S8C**; Preiner et al., 2024). This higher magnitude is consistent with a higher stress severity in W-treated plants (**Supplementary Tables 8A, B**) and suggests that, while both elements trigger the accumulation of protective metabolites, levels of these metabolites not only differ between the modes of nitrogen nutrition but also scale with the severity of the metabolic disruption (**Supplementary Table 8C**). While these compounds have been reported to accumulate in response to various heavy metals and are well-established markers of HM stress, the exact mechanism of their protective function is still not fully understood (Menéndez et al., 2019; Spormann et al., 2023; Szabados and Savouré, 2010). Similarly, it is difficult to infer the exact role these secondary compounds play under excess molybdenum and tungsten, and whether these compounds are produced as an active stress response or because of the metabolic disturbances. N fix soybean plants treated with 0.5 mM tungsten not only showed increased levels of non-enzymatic antioxidants, but also a significant accumulation of various antioxidative defense enzymes such as peroxidases, glutathione peroxidase, glutathione reductase, superoxide dismutase (Cu–Zn), and H-type thioredoxin (Preiner et al., 2024). In line with this, the observed increase in protective compounds under excess Mo conditions may thus indicate an increased oxidative stress response mechanism in N fix plants. This distinct antioxidative response suggests that, at least at the levels tested, the metabolic disturbances caused by W were more severe and triggered a much broader and more extensive enzymatic antioxidative response. However, as previously discussed, the observed significant increase of these protective compounds could also be seen as a consequence of the observed disruption of primary metabolic processes and hampered growth, as the plant sequesters excess nitrogen and balances C/N levels (Balestrasse et al., 2005; Paschalidis et al., 2019). In the case of tungsten, we hypothesized that the symbiont-dependent enhanced production of these protective compounds contributed to the observed immobilization of W in the root system, which, together with enhanced enzymatic-antioxidant defense, may have contributed to the maintenance of root and shoot length in symbiotic soybean plants (N fix), whereas shoot length was significantly reduced in nitrogen-fertilized plants (N fed) (**Supplementary Tables 8A, C**; Preiner et al., 2024). In line with this, N fix leaves showed a significant increase in phenolic compounds under W stress, accompanied by a lower, though non-significant, IC50 value, indicating higher antioxidant capacity (**Supplementary Table 8C**; Preiner et al., 2024). Yet, the hypothesis that the accumulation of protective compounds is driven by a symbiosis-mediated active metabolic readjustment does not necessarily exclude the idea that it represents a consequence of the overall metabolic disturbances and significantly hampered growth observed in N fix plants under both Mo and W stress (**Supplementary Tables 8A, B**). This is consistent with the findings of Balestrasse et al. (2005), who observed that similar changes in the levels of putrescine, spermidine and proline in roots and nodules of soybean plants subjected to various Cd^2+^ concentrations did not result in enhanced stress resilience.

The notion that Mo and W elicit qualitatively similar but quantitatively distinct responses is also evident when comparing the proteomic data (**Figure 5**; **Supplementary Table 6**). As described above, leaves of both treatments showed a loss of iron-buffering capacity (**Supplementary Figure 6**, **7**), which potentially led to an increase in free iron-induced oxidative stress. Under excess Mo conditions, this was accompanied by a decrease in various important components of the photosynthetic machinery (RuBisCO large subunit, geranylgeranyl reductase/CHL P, chloroplastic heme oxygenase, plastid ribosomal and biosynthetic enzymes), consistent with the observed impaired photosynthetic efficiency (**Supplementary Table 8B**). In leaves of W-stressed N fix plants, conversely, proteins involved in oxidative- and nitrogen-stress management accumulated (**Figure 5B**; **Supplementary Table 6A**). In roots (**Figure 5E**; **Supplementary Table 6C**), both metals induced a broad set of predominantly defensive proteins, including pathogenesis-related proteins (chitinases, thaumatin-like/PR-5, PR-1, Gnk2-homologous protein), redox and cell-wall modifying enzymes (class III peroxidases, FAD-binding oxidoreductases) (Shigeto and Tsutsumi, 2016), as well as protease and α-amylase/subtilisin inhibitors. This defensive response was more extensive under W stress, where various enzymes involved in cell-wall reinforcement and remodeling (BURP-domain protein, germin-like protein, pectinesterase inhibitor) additionally accumulated, which is consistent with our observation of W immobilization in the root system (Bernier and Berna, 2001; Krzesłowska, 2011; Wang et al., 2012). Taken together, our proteomic data point to the disruption of iron homeostasis and oxidative stress as key drivers of Mo- and W-induced stress. At the whole-plant level, however, the two metals appear to induce distinct strategies. While under W stress, N fix plants showed increased investment in cell-wall stiffening as well as prevention of protein degradation in roots (**Figure 5E**), Mo-treated plants instead downregulated photosynthesis and modulated protein biosynthesis in leaves (NAC-A/B domain-containing protein, 60S ribosomal protein L22-2) potentially to prevent further oxidative damage (**Figure 5B**; **Supplementary Table 6A**). Both strategies appear to have come at direct cost to growth and photosynthetic function and provide an explanation as to why the observed accumulation of protective compounds did not result in restored growth.

Another explanation for the different magnitude of the observed protective metabolite response between Mo- and W-induced stress may lie in the readjustment of nitrogen metabolism in N fix plants. The above discussed accumulation of N-metabolism intermediaries (allantoin, allantoic acid, glutamine and glutamate) and various proteases and protease inhibitory proteins under excess Mo resembles the N-metabolic disturbances previously reported under W stress (**Supplementary Table 8E**; Preiner et al., 2024). W-treated N fix roots, however, additionally exhibited a ten-fold increase in glutamate dehydrogenase and a three-fold increase in uricase abundance as well as other enzymes involved in nitrogen metabolism (**Supplementary Table 8D**, Preiner et al., 2024), consistent with an attempt to reroute excess nitrogen via alternative assimilatory and catabolic pathways (Balestrasse et al., 2004). This more extensive metabolic readjustment under W may explain the substantially higher levels of proline relative to Mo-treated plants (**Supplementary Table 8C**). As W is a known inhibitor of Moco containing enzymes (Adamakis et al., 2012), and given the previously demonstrated reduction of nitrate reductase (NR) activity in W-stressed soybeans (Preiner et al., 2019), W may also have affected key enzymes involved in ureide biosynthesis downstream of nitrogenase mediated N_2_ fixation, such as xanthine dehydrogenase. In contrast, excess Mo would not be expected to inactivate these enzymes and, as discussed above, potentially even has a mild fertilizing effect, as was already reported for excess Mo in pea plants (Kevresan et al., 2001). This is also reflected in the abundances of nitrogen-assimilatory enzymes of N fed plants. Although NR activity was not directly measured in the present study, nitrite reductase (NiR) abundance was almost four-fold lower and glutamine synthetase abundance three-fold lower in roots of W-treated N fed plants (**Supplementary Table 8D**; Preiner et al., 2024). Under excess Mo, these enzymes were also reduced but far less than under W, and glutamate synthase abundance was even increased (**Supplementary Tables 6A, C**). This may account for the milder N-metabolic disruption (**Supplementary Tables 8D, E**) and the absence of a significant effect on plant biomass or growth observed in N fed plants under excess Mo (**Table 1**, **Supplementary Table 8A**). Under W stress, by contrast, the observed significant reduction of shoot length in N fed plants was consistent with the known inhibitory effect of W on nitrate reduction (**Supplementary Table 8A**; Preiner et al., 2019, 2024). This difference supports the idea that while W-substituted molybdoenzymes such as nitrate reductase are catalytically inactive, excess Mo presumably does not affect enzyme function in the same way.

For N fix plants, the considerably higher Mo uptake (**Table 2**), conversely, resulted in an additional metabolic burden, as maintaining the oxygen-sensitive nitrogenase enzyme under excess Mo required a greater allocation of resources to antioxidative defense, as evidenced by the significantly increased levels of flavonoids and phenolic compounds in roots (**Figure 2**; **Supplementary Table 8C**). Additionally, this higher Mo uptake disrupted nutrient homeostasis (**Table 2**) and biosynthetic sinks for fixed nitrogen, leading to an accumulation of N-compounds in leaves and roots (**Figure 4**, **Supplementary Figure 4**; **Supplementary Table 8E**), which, as discussed above, potentially resulted in a feedback inhibition of N_2_ fixation. This may explain why symbiotic plants, despite exhibiting a stronger protective response, were more sensitive to excess Mo than N fed plants. Overall, the divergent outcomes under Mo and W stress appear to have been shaped primarily by the distinct biological roles of the two metals under the two modes of nitrogen nutrition, rather than the observed protective response, with W acting as an inhibitor of molybdopterin-cofactor enzymes and Mo as an essential nutrient.

## 5 Conclusion

In conclusion, the stress response observed in soybean plants grown under excess concentrations of the essential plant nutrient Mo shares several hallmarks with that previously described for its chemical analog W (Preiner et al., 2024). The metabolic response to both elements was strongly dependent on the mode of N nutrition, as symbiotically grown plants (N fix) were not only more sensitive to excess levels of Mo and W but also showed a stronger metabolic readjustment and a greater capacity to produce protective compounds. Both Mo and W led to a disruption of Fe homeostasis and reduced ferritin abundances, as well as an accumulation of soluble carbohydrates, flavonoids, phenolics, polyamines, proline, gluconic acid and nitrogenous compounds in N fix plants. In both cases, however, this enhanced production of protective compounds did not translate to a reduced stress severity in N fix plants. This convergent response is consistent with the chemical similarity between MoO_4_^2−^ and WO_4_^2−^, which likely resulted in a partial overlap regarding affected metabolic pathways, cofactor interactions and intracellular fate.

Nevertheless, clear differences in overall severity of the metabolic disruption as well as the induced stress response were apparent between the Mo- and W-treated plants. Although the overall severity of metabolic disruption was greater under W stress, N fix plants were more sensitive to Mo compared to nitrogen-fertilized plants (N fed). This is consistent with the different biological roles of Mo and W. While W-substituted molybdoenzymes are catalytically inactive, excess Mo might have even enhanced certain Mo-dependent enzymatic functions in N fed plants.

To better understand how the rhizobia symbiosis shapes the plant’s response to Mo- and W-induced stress, future work should focus on the temporal and dose-dependent dynamics of the stress response in nodule tissue as well as at the symbiosome membrane. In particular, the key interface between plant host and symbiont deserves further investigation to complete the picture with information on initial stress sensing, uptake processes, signaling, and direct effects on nitrogen fixation and assimilation processes.

## Data Availability

The datasets presented in this study can be found in online repositories. The names of the repository/repositories and accession number(s) can be found below: http://www.proteomexchange.org/, PXD047532.
