# Supplementary material for: Excess molybdenum impairs growth, nitrogen metabolism and nutrient translocation of soybean in Bradyrhizobium symbiosis, with parallels to tungsten stress
Source: Front Plant Sci. 2026 Aug 7;17:1905971. doi: 10.3389/fpls.2026.1905971 (PMC13493601; doi:10.3389/fpls.2026.1905971)
Supplement: Supplementary file 1 [file DataSheet1.pdf]

Supplementary Material

Excess Molybdenum Impairs Growth, Nitrogen Metabolism and Nutrient Translocation of Soybean in *Bradyrhizobium* Symbiosis, with Parallels to Tungsten Stress

Julian Preiner[1], Irene Steccari[1], Eva Oburger[2], Stefanie Wienkoop[*1],

[1] Division of Molecular Systems Biology, Department of Ecogenomics and Systems Biology, University of Vienna, Vienna, Austria

[2] Institute of Soil Research, Department of Forest and Soil Sciences, University of Natural Resources and Life Sciences Vienna, Tulln, Austria

*** Correspondence:**
Stefanie Wienkoop
stefanie.wienkoop@univie.ac.at

Supplementary Figure 1 | Photos of soybean roots (*Glycine max* cv. Primus) grown semi-hydroponically without (Control) or with molybdenum (0.5 mM Mo) and two different nitrogen supply regimes (N fix: inoculated with *B. japonicum*, week 1&2 0.25mM KNO_3 & week 3–5 zero N; N fed: week 1–5 10mM KNO_3). Brightness was increased by +40% in all pictures, pictures of whole plants on the table were also sharpened by +50%. For better comparability pictures were resized and a graphical representation of the scale was added. Each square represents 1 cm.

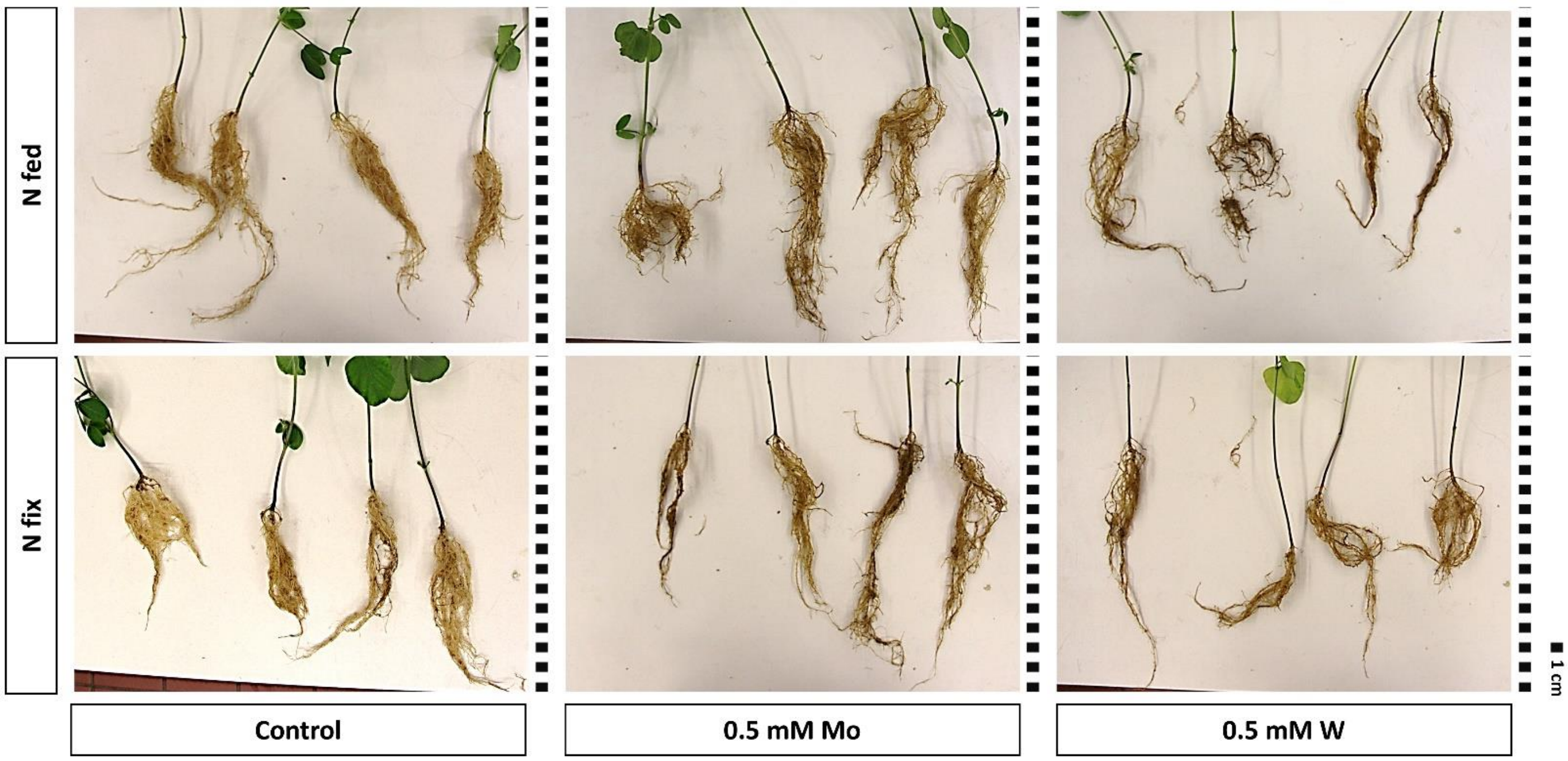

Supplementary Figure 2 | (A) Non-photochemical quenching (NPQ), (B) relative electron transfer rate (ETR), (C) Effective photochemical quantum yield (Y(II)) and (D) photochemical fluorescence quenching coefficient (qP) for PSII of dark-adapted soybean plants during the rapid light curve (RLC) measurement. Values are means ± SE (n=4). Lines were smoothed by loess method; gray shades indicate 95% confidence interval. Plants (*Glycine max* cv. Primus) were grown semi-hydroponically without (Ctrl.) or with 0.5 mM molybdenum (Mo) in the nutrient solution and two different nitrogen supply regimes (N fix: inoculated with *B. japonicum*, week 1&2 0.25 mM KNO_3 & week 3–5 zero N; N fed: week 1–5 10 mM KNO_3).

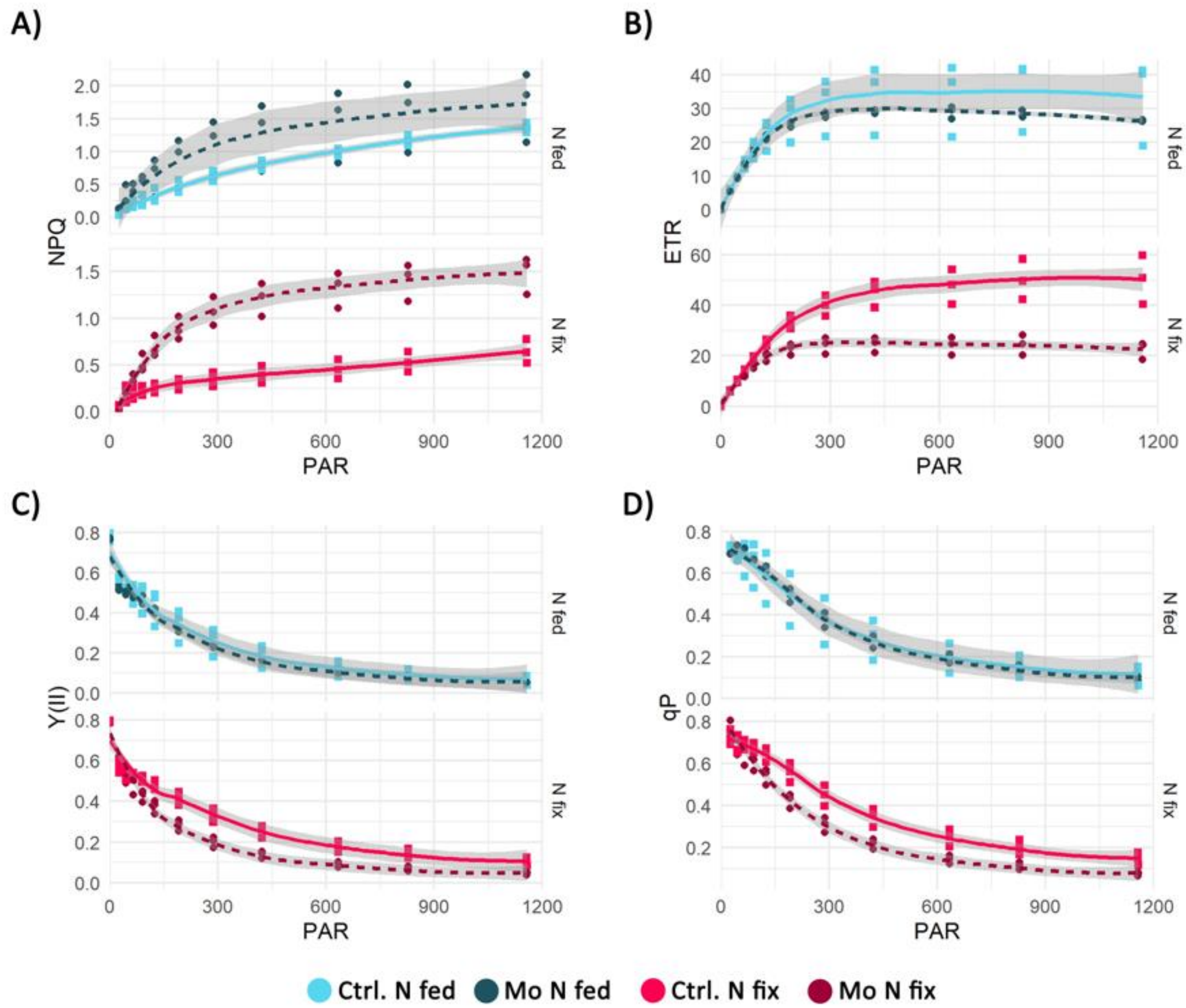

Supplementary Figure 3 | PCA loadings (PC1/PC2) of leaf (A) and root (B) PCA including log^{2} transformed data including metabolite concentrations, LFQ intensities of significantly changed proteins (n=3–4, ANOVA, post hoc Tukey, $p < 0.05$). Figure shows a cutoff for PC1 and PC2 at ±0.065 (dark grey) as well as at ±0.075 (dashed line)

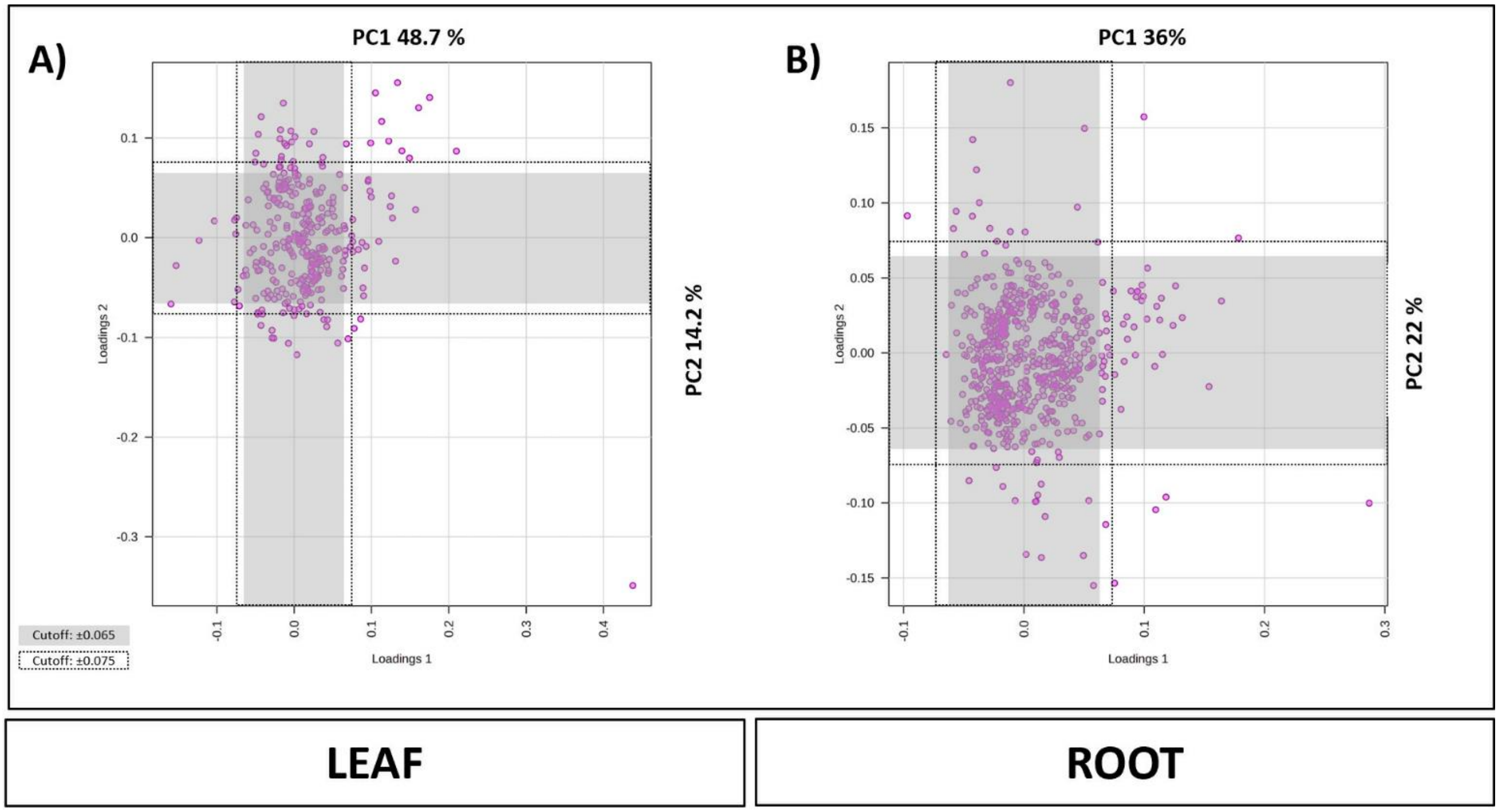

Supplementary Figure 4 | Heatmap of leaf and root metabolites and proteins with PC2 loadings higher than ±0.65. Soybean plants (*Glycine max* cv. Primus) were grown semi-hydroponically without (Ctrl.) or with 0.5 mM molybdenum (Mo) in the nutrient solution and two different nitrogen supply regimes (N fix: inoculated with *B. japonicum*, week 1&2 0.25 mM KNO_3 & week 3–5 zero N; N fed: week 1–5 10 mM KNO_3). Colors represent z-transformed mean values (n=3–4). Letters indicate significant differences between the different molybdenum treatments and nitrogen regimes (ANOVA, post hoc DGC, $p < 0.05$).

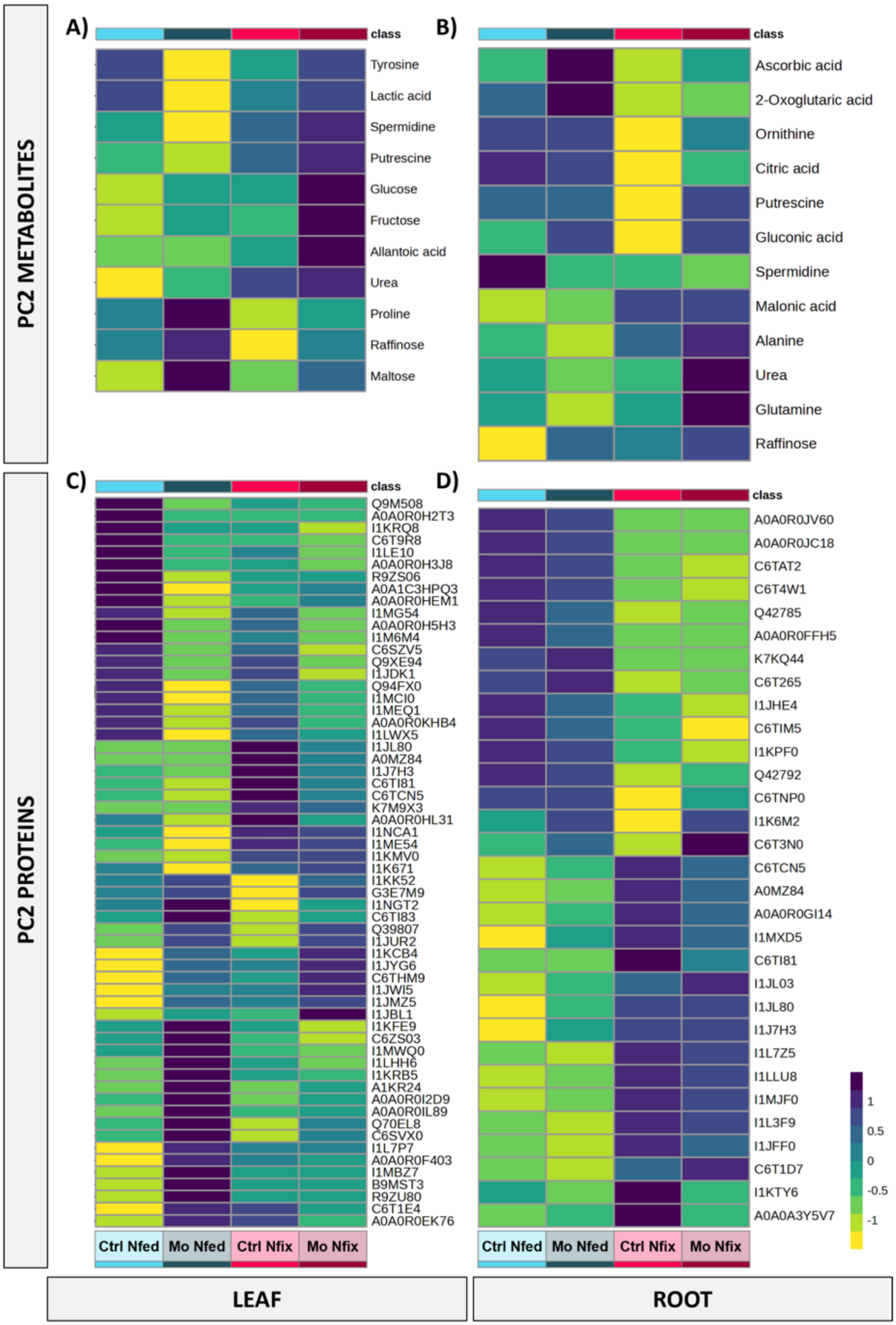

Supplementary Figure 5 | Venn Diagrams of significantly changed leaf and root proteins. Soy bean plants (*Glycine max* cv. Primus) were grown semi-hydroponically without (Ctrl.) or with 0.5 mM molybdenum (Mo) in the nutrient solution and two different nitrogen supply regimes (N fix: inoculated with B. japonicum, week 1&2 0.25 mM KNO_3 & week 3–5 zero N; N fed: week 1–5 10 mM KNO_3).

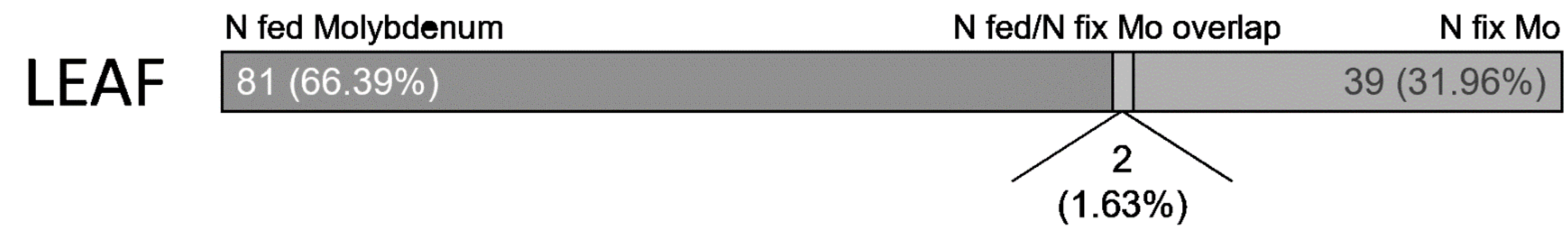

Supplementary Figure 6 | GO enrichment analysis of significantly changed leaf and root proteins under excess molybdenum and tungsten treatment. Bubble plot showing the results of GO enrichment analysis of significantly changed leaf proteins with the highest PC2 loadings (±0.075) and significantly changed root proteins with the highest PC1 loadings (±0.065), performed with ShinyGO 0.82 (Ge et al., 2020) and the GO Ontology database (DOI: 10.5281/zenodo.10536401, released 2024-01-17) for *Glycine max* using the default settings. Enrichment analysis was performed for biological processes (BP) and molecular functions (MF). Fisher's exact test was used as the statistical test, and the false discovery rate (FDR) was calculated for significant enrichments. Detailed results can be found in Supplementary Table 7. The plot shows $-\log_{10}$-transformed FDR p-values; bubble size represents gene count and color indicates fold enrichment. Soybean plants (*Glycine max* cv. Primus) were grown semi-hydroponically without (Ctrl.) or with 0.5 mM molybdenum (Mo) or tungsten (W) in the nutrient solution and under two different nitrogen supply regimes (N fix: inoculated with *B. japonicum*, weeks 1 & 2 0.25 mM KNO_3 and weeks 3–5 zero N; N fed: weeks 1–5 10 mM KNO_3).

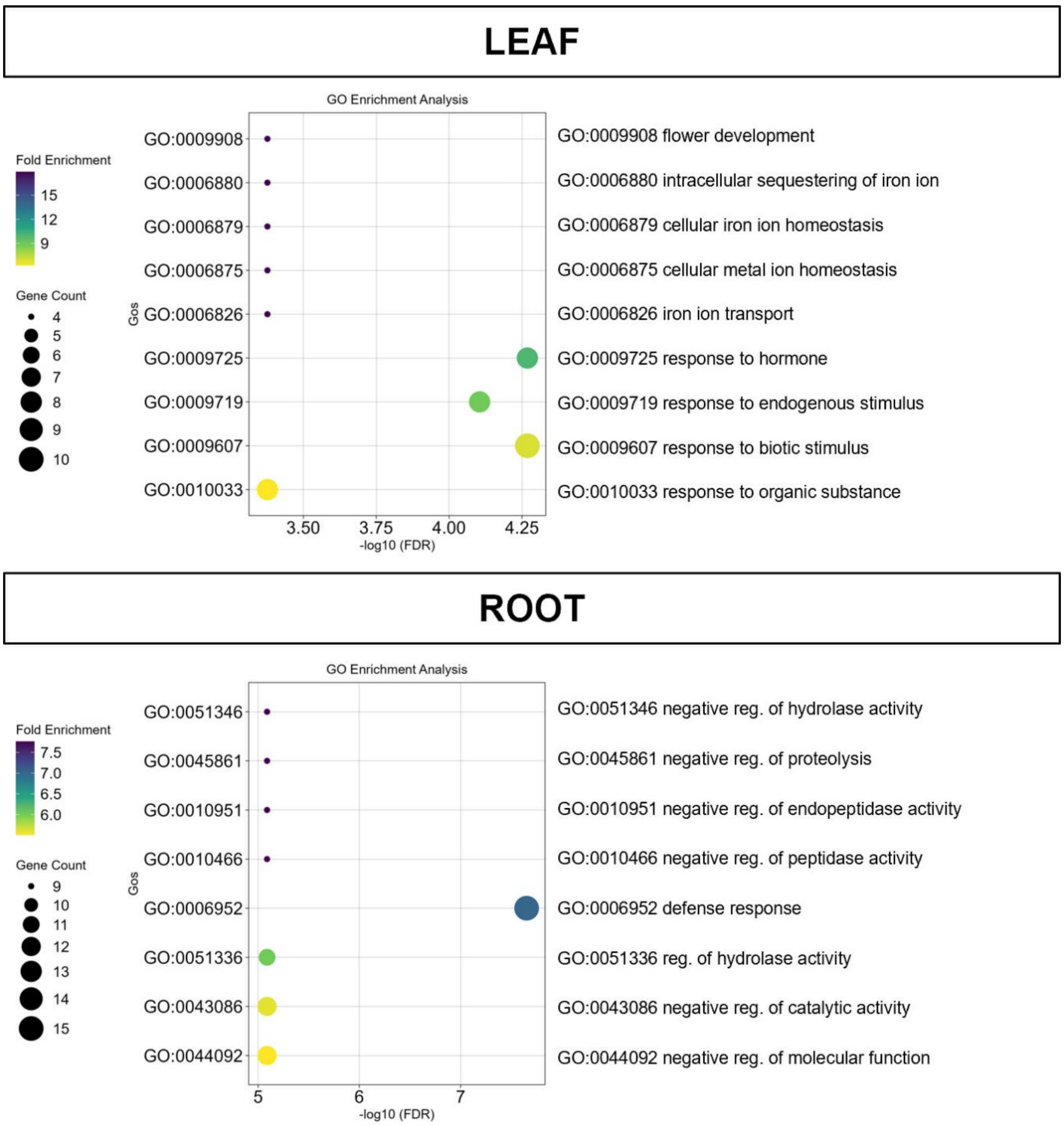

SI FIGURE 7 | Abundance of non-haem ferritins in leaves and roots of soybean under molybdenum treatment. Bar chart showing LFQ intensities of significantly changed non-haem ferritins in leaves and roots of soybean (*Glycine max* cv. Primus), comparing controls and Mo treatments in both nitrogen regimes. Soybean plants were grown semi-hydroponically without (Ctrl.) or with 0.5 mM molybdenum (Mo) in the nutrient solution and under two different nitrogen supply regimes (N fix: inoculated with *B. japonicum*, weeks 1 & 2 0.25 mM KNO_3 and weeks 3–5 zero N; N fed: weeks 1–5 10 mM KNO_3). Values are means ± SE (*n*=3–4). Asterisks indicate significant differences between control and Mo treatment (ANOVA, post hoc Tukey HSD, $p < 0.05$).

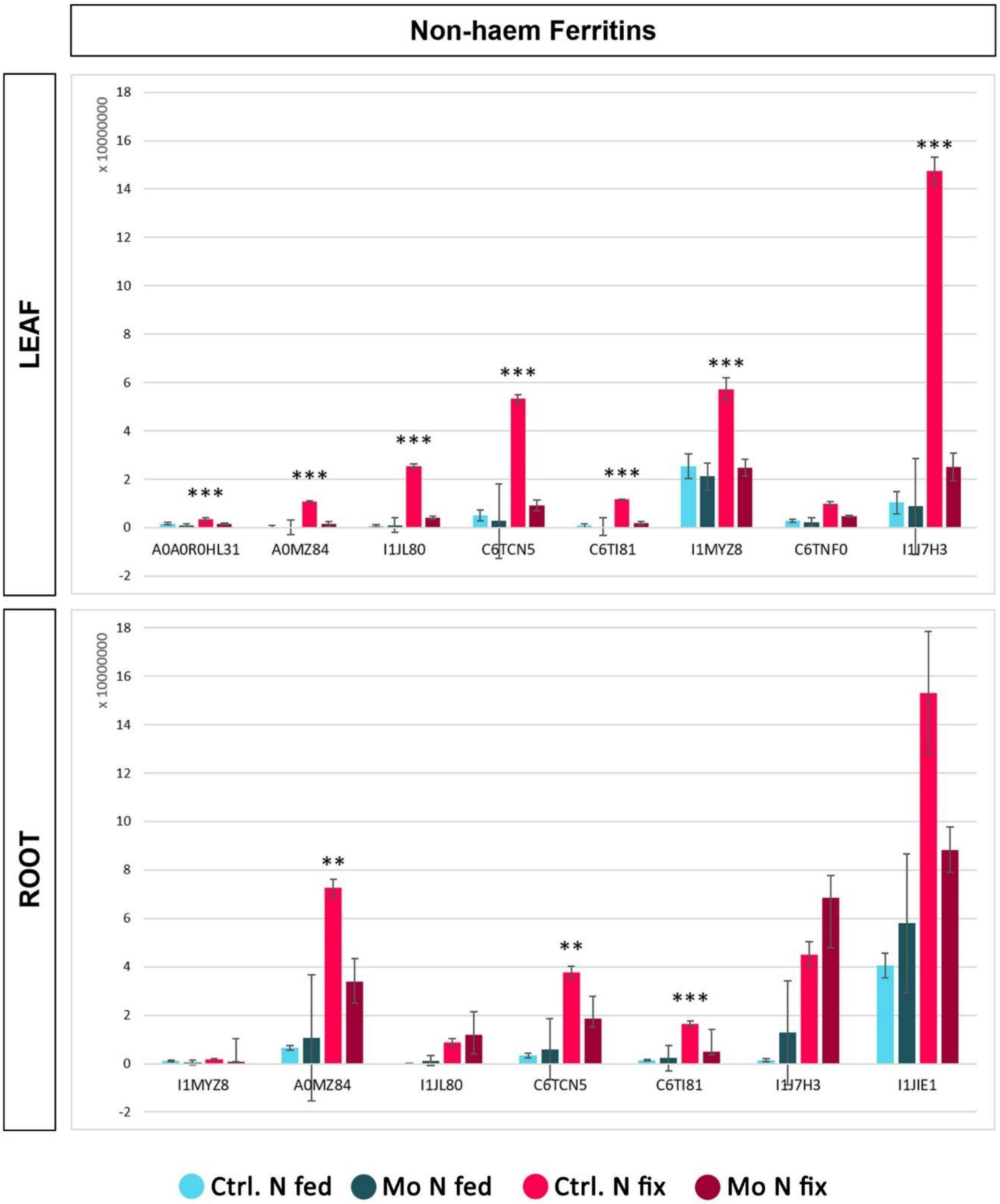

Supplementary Table 1| Concentrations of photosynthetic pigments (A) as well as chlorophyll fluorescence parameters (B) of dark-adapted soybean plants during the rapid light curve (RLC) measurement at a PAR of 191, 634 and 1157. Parameters shown are Fv/Fm potential quantum yield, Y(II) effective photochemical quantum yield, ETR relative electron transport rate, NPQ non-photochemical quenching, qP coefficient of photochemical quenching and qN coefficient of non-photochemical quenching. Plants (*Glycine max* cv. Primus) were grown semi-hydroponically without (Control) or with 0.5 mM molybdenum (Mo) in the nutrient solution and two different nitrogen supply regimes (N fix: inoculated with *B. japonicum*, week 1&2 0.25 mM KNO_3 & week 3–5 zero N; N fed: week 1–5 10 mM KNO_3). Values are means ± SE (n=4). Letters indicate significant differences between the different molybdenum treatments and nitrogen regimes (ANOVA, post hoc DGC, $p < 0.05$).

Supplementary Table 2 | Leaf tissue concentrations of sugars and sugar alcohols (A), amino acids (B), organic acids (C), polyamines (D) as well as further "non-QC metabolites" (E) of soybean (*Glycine max* v. Primus) grown semi-hydroponically without (Control) or with 0.5 mM molybdenum in the nutrient solution and two different nitrogen supply regimes (N fix: inoculated with *B. japonicum*, week 1&2 0.25mM KNO_3 & week 3–5 zero N; N fed: week 1–5 10mM KNO_3). Values are means ± SE (n=3–4). Letters indicate significant differences between the different molybdenum treatments and nitrogen regimes (ANOVA, post hoc DGC, $p < 0.05$). PCA loadings for metabolites are provided for PC1–PC4.

Supplementary Table 3 | Root tissue concentrations of sugars and sugar alcohols (A), amino acids (B), organic acids (C), polyamines (D) as well as further "non-QC metabolites" (E) of soybean (*Glycine max* v. Primus) grown semi-hydroponically without (Control) or with 0.5 mM molybdenum in the nutrient solution and two different nitrogen supply regimes (N fix: inoculated with *B. japonicum*, week 1&2 0.25mM KNO_3 & week 3–5 zero N; N fed: week 1–5 10mM KNO_3). Values are means ± SE (n=3-4). Letters indicate significant differences between the different molybdenum treatments and nitrogen regimes (ANOVA, post hoc DGC, $p < 0.05$). PCA loadings for metabolites are provided for PC1–PC4.

Supplementary Table 4 | List of significantly changed leaf proteins between control and molybdenum (0.5 mM Mo, supplied as sodium tungstate) of soybean (*Glycine max* v. Primus) grown semi-hydroponically without (Control) or with 0.5 mM molybdenum in the nutrient solution and two different nitrogen supply regimes (N fix: inoculated with *B. japonicum*, week 1&2 0.25mM KNO_3 & week 3–5 zero N; N fed: week 1–5 10mM KNO_3). Values are z-transformed means (n=3–4). Asterisks indicate significant difference (ANOVA, Post Hoc Tukey, $p<0.05$). Proteins uniquely identified by prototypic peptides are indicated with an x.

Supplementary Table 5 | List of significantly changed root proteins between control and molybdenum (0.5 mM Mo, supplied as sodium tungstate) of soybean (*Glycine max* v. Primus) grown semi-hydroponically without (Control) or with 0.5 mM molybdenum in the nutrient solution and two different nitrogen supply regimes (N fix: inoculated with *B. japonicum*, week 1&2 0.25mM KNO_3 & week 3–5 zero N; N fed: week 1–5 10mM KNO_3). Values are z-transformed means (n=3–4). Asterisks indicate significant difference (ANOVA, Post Hoc Tukey, $p<0.05$). Proteins uniquely identified by prototypic peptides are indicated with an x.

Supplementary Table 6 | Detailed statistics for proteins and metabolites depicted in heatmaps in Figure 5. (A) shows leaf proteins and (B) leaf metabolites with PC2 loadings ≥± 0.075 that were significantly changed in the excess Mo, W or both treatments. (C) shows root proteins and (D) root metabolites with PC1 loadings ≥± 0.065 that were significantly changed in the excess Mo, W or both treatments. Soybean plants (*Glycine max* cv. Primus) were grown semi-hydroponically without (Ctrl.) or with 0.5 mM molybdenum (Mo) or tungsten (W) in the nutrient solution and two different nitrogen supply regimes (N fix: inoculated with *B. japonicum*, week 1&2 0.25 mM KNO3 & week 3–5 zero N; N fed: week 1–5 10 mM KNO3). Values are mean LFQ intensities ± SE (n = 3–4). Letters and Asterisks indicate significant difference (ANOVA, Post Hoc Tukey, $p<0.05$).

Supplementary Table 7 | Results of GO enrichment analysis of significantly changed leaf proteins with highest PC2 loadings (±0.075) as well as significantly changed root proteins with highest PC1 loadings (±0.065) performed with ShinyGO 0.82 (Ge et al., 2020) and GO Ontology database (DOI:10.5281/zenodo.10536401 released 2024-01-17) for Glycine max (Aleksander et al., 2026; Ashburner et al., 2000) using the default settings. Soybean plants (*Glycine max* cv. Primus) were grown semi-hydroponically without (Ctrl.) or with 0.5 mM molybdenum (Mo) or tungsten (W) in the nutrient solution and two different nitrogen supply regimes (N fix: inoculated with *B. japonicum*, week 1&2 0.25 mM KNO3 & week 3–5 zero N; N fed: week 1–5 10 mM KNO3). Enrichment analysis was performed for biological process (BP) and molecular functions (MF). As statistical test Fisher's exact and false discovery rate (FDR) was calculated for significant enrichments.

Supplementary Table 8 | Detailed statistics for parameters used to discuss stress severity and sensitivity as well as nitrogen metabolism (biomass, photosynthetic markers, protective compounds, N-metabolism proteins and N-metabolism metabolites) between Mo- and W-treated plants. Soybean (*Glycine max* cv. Primus) were grown semi-hydroponically without (Control) or with 0.5 mM molybdenum (Mo) or tungsten (W) and two different nitrogen supply regimes (N fix: inoculated with *B. japonicum*, week 1&2 0.25 mM KNO_3 & week 3–5 zero N; N fed: week 1–5 10 mM KNO_3). **(A)** Shoot (a) and root (b) length (cm), fresh weight (g), dry weight (mg), nodule count, nodule weight as well as total soluble protein concentrations ($\mu g\ mg^{-1}$ FW). Values are means ± SE (biomass n = 4, protein content n = 3–4). Letters indicate significant differences between the different molybdenum treatments and nitrogen regimes (ANOVA, post hoc DGC, $p < 0.05$). For nodule weight and nodule count, statistical analysis was not performed for nitrogen fertilized-plants (N fed) due to insufficient replication ($n \leq 2$); data points represent individual values or the mean of available replicates where applicable. Standard errors and significance could not be calculated and are marked as N.D. (Not determined). **(B)** Concentrations of photosynthetic pigments (a) as well as chlorophyll fluorescence parameters (b) of dark-adapted soybean plants during the rapid light curve (RLC) measurement at a PAR of 191, 634 and 1157. Parameters shown are Fv/Fm potential quantum yield, Y(II) effective photochemical quantum yield, ETR relative electron transport rate, NPQ non-photochemical quenching, qP coefficient of photochemical quenching and qN coefficient of non-photochemical quenching. Values are means ± SE (n = 4). Letters indicate significant differences between the different molybdenum treatments and nitrogen regimes (ANOVA, post hoc DGC, $p < 0.05$). **(C)** Leaf (a) and root (b) tissue concentrations of protective compounds (Tannins, Phenolic compounds, Flavonoids, IC50 value, Putrescine, Spermidine, Proline, Gluconic acid). Values are means ± SE (n = 3–4). Letters indicate significant differences between the different molybdenum treatments and nitrogen regimes (ANOVA, post hoc DGC, $p < 0.05$). **(D)** List of N-metabolism associated root proteins. Values are mean LFQ intensities (n = 3–4). Asterisks indicate significant difference (ANOVA, Post Hoc Tukey, $p<0.05$). **(E)** Leaf (a) and root (b) tissue concentrations of N-metabolism intermediary compounds (Allantoic acid, Allantoin, Asparagine, Glutamic acid, Glutamine, Urea). Values are means ± SE (n = 3–4). Letters indicate significant differences between the different molybdenum treatments and nitrogen regimes (ANOVA, post hoc DGC, $p < 0.05$).
